# Ketosis suppression and ageing (KetoSAge): the effect of suppressing ketosis on SHBG and sex hormone profiles in healthy premenopausal women, and its implications for cancer risk and therapy

**DOI:** 10.3389/fnut.2025.1731915

**Published:** 2026-01-02

**Authors:** Isabella D. Cooper, Lucy Petagine, Adrian Soto-Mota, Tomás Duraj, Thomas N. Seyfried, Derek C. Lee, Naja Cooper, Yvoni Kyriakidou

**Affiliations:** 1 Metabolic Endocrine Cancer Cardiovascular and Ageing Research, Centre for Nutraceuticals, School of Life Sciences, University of Westminster, London, United Kingdom; 2 Neuroscience, Inflammatory Disorders and Therapeutics Group, Research Centre for Nutraceuticals, School of Life Sciences, University of Westminster, London, United Kingdom; 3 Unidad de Investigación en Enfermedades Metabólicas, Instituto Nacional de Ciencias Médicas y Nutrición Salvador Zubirán, Mexico City, Mexico; 4 Tecnologico de Monterrey, School of Medicine, Mexico City, Mexico; 5 Biology Department, Boston College, Chestnut Hill, MA, United States; 6 Faculty of Science and Engineering, Queen Mary University of London, London, United Kingdom

**Keywords:** ageing, BHB, GKI, HOMA-IR, hyperinsulinaemia, insulin resistance, ketosis, oestrogen

## Abstract

**Introduction:**

Insulin resistance and hyperinsulinaemia significantly influence female hormone regulation and reproductive health. Despite increasing research, the complex pathways by which nutritional and metabolic signals regulate reproductive function remain poorly understood. Sex hormone-binding globulin (SHBG) is a key protein whose function is modulated by hyperinsulinaemia, liver function, and metabolic status, thereby influencing the active signalling of circulating sex steroids and intracellular signalling, which in turn, impacts endocrine and reproductive physiology. Consequently, SHBG serves as a valuable biomarker for understanding the metabolic-hormonal interactions within the endocrine axis. Ketogenic diets have demonstrated efficacy in reversing insulin resistance, resolving markers of liver disease, and improving metabolic health. In this study, we investigated the impact of suppressing ketosis (hypoketonaemia) on biomarkers of female reproductive and endocrine function in the Ketosis Suppression and Ageing cohort.

**Methods:**

Ten lean (BMI, 20.52 kg/m^2^ ± 1.39), healthy, premenopausal women (mean age, 32.30 ± 8.97 years), who maintained nutritional ketosis for an average of 3.9 years (± 2.3), participated in a three-phase intervention trial: 21-days of baseline data-collection in euketonaemia, 21-days of hypoketonaemia, and 21-days return to euketonaemia.

**Results:**

Suppression of ketosis resulted in a significant 0.67-fold decrease in SHBG levels (*p* = 0.0015). SHBG was significantly and inversely associated with insulin (*p* = 0.0010), insulin resistance score (HOMA-IR; *p* = 0.0012), glucose ketone index (GKI; *p* = 0.0183), leptin (*p* = 0.0016), insulin-like growth factor-1 (IGF-1; *p* = 0.0172), free T3 (*p* = 0.0001), and gamma-glutamyl transferase (GGT; *p* = 0.0024). A significant positive association between SHBG and GLP-1 (*p* = 0.0295) was observed. Menstrual cycle phase was a statistically significant predictor of follicle-stimulating hormone (FSH) levels, with higher FSH levels during ovulation than during the follicular phase (*p* = 0.0097).

**Discussion:**

SHBG is a sensitive biomarker of metabolic-endocrine status, with broader implications for cancer, and reproductive function. Chronic hypoketonaemia negatively affects SHBG production and hormonal balance. The implications of sex-hormone regulation for cancer prevention and therapy are discussed.

## 1 Introduction

In recent years, there has been growing interest in the interplay between metabolic dysfunction and female-specific health conditions, including hormone-related cancers and polycystic ovary syndrome (PCOS). PCOS is a prevalent endocrine disorder affecting women of reproductive age and is frequently associated with metabolic disturbances, including obesity, insulin resistance (IR), and elevated androgen levels (hyperandrogenism) (1–3). Chronic hyperinsulinaemia, a hallmark of modern lifestyle-related metabolic factors, has been identified as a central driver in the development of oestrogen-dominant pathologies, such as breast, endometrial, and ovarian cancers, as well as PCOS (4–15). These conditions often coexist with features of metabolic syndrome (MetS), highlighting the intricate link between reproductive and metabolic health in women. Chronically elevated basal levels of insulin, i.e., hyperinsulinaemia, suppresses ketogenesis, which is detectable as *hypoketonaemia*, where beta-hydroxybutyrate (BHB) ketone body concentrations are for several consecutive days consistently < 0.5 mmol/L before the evening meal at least three hours post-prandially (16, 17).

Sex hormones, particularly oestrogens, androgens, and progesterone, play a significant role in regulating insulin sensitivity and metabolic function, including processes related to energy balance and inflammatory pathways (18). Oestrogen exerts protective effects on metabolic regulation, especially in glucose homeostasis and insulin sensitivity, and modulates immune cell activation and function through multiple mechanisms. Reduced oestrogen levels, commonly observed during menopause and in certain hormonal disorders, have been associated with an increased risk for type 2 diabetes (T2DM) and cardiovascular disease (CVD).

Oestrogen and testosterone circulate in the bloodstream primarily bound to carrier proteins, most notably the liver-synthesised glycoprotein sex hormone-binding globulin (SHBG) and albumin. SHBG is a key regulator of the bioavailability of circulating sex steroids, and its serum concentrations modulated by both hormonal and metabolic factors, especially insulin (1, 19). Emerging evidence suggests that the balance between testosterone and oestrogen may be associated with carbohydrate metabolism and insulin (20). Within this context, lower circulating SHBG concentrations are often considered indicative of a more androgenic hormonal environment. It has been proposed that observed associations between SHBG and glycaemic markers (i.e., insulin, fasting glucose or HOMA-IR) may reflect the underlying steroid milieu, rather than a direct metabolic function of SHBG itself (21). Additionally, decreased SHBG levels have been linked to an increased risk of developing MetS (22), and show inverse correlations with body mass index (BMI) and waist circumference (1). Supporting these findings, cross-sectional studies in both men and women have demonstrated that higher SHBG levels correlate with a lower risk of T2DM, with robust associations observed in women (20). Lower levels of SHBG are also associated with increased incidence of cancer (13, 14, 23, 24). Despite its critical regulatory function, SHBG is rarely measured in clinical practice, even though it plays an essential role in the modulation of sex hormone bioavailability and intracellular signalling modulation. Many cancers, including breast, endometrial, ovarian, prostate, pancreatic, lung and brain tumours are classified as sex hormone-sensitive malignancies (13–15, 25–28). Alterations in SHBG concentrations can therefore affect not only the circulating levels of active sex steroids but also their signalling activity within target tissues, influencing cancer susceptibility and progression.

Recent clinical studies have also highlighted the impact of dietary carbohydrate restriction, particularly ketogenic diets, on SHBG concentrations and metabolic function in women. In both PCOS and non-PCOS populations, *euketonaemia* (BHB ≥ 0.5 mmol/L - < 5 mmol/L) interventions have been associated with increased SHBG levels, alongside improvements in insulin sensitivity, glycaemic control, and androgen profiles (16–18, 29–35). These effects are mediated by reductions in serum basal insulin, whereas higher levels of insulin that suppress ketogenesis causing hypoketonaemia, and concomitantly inhibit hepatic SHBG synthesis (36). Consequently, SHBG serves as a sensitive biomarker of metabolic-endocrine status, further refining clinicians’ and research scientists’ ability to detect subclinical hyperinsulinaemia (SCHI) and, therefore, the associated increased risk for certain cancers, cardiovascular and neurodegenerative diseases. Earlier detection of SCHI would enable earlier intervention, preventing the development of a serious health condition.

There are limitations with the use of fasting glycaemic and insulin markers. Hyperglycaemia may remain undetected when relying solely on haemoglobin A1c (HbA1c) or fasting glucose measurements, as compensatory hyperinsulinaemia suppresses fasting glucose and masks underlying pathology. In such cases, persistent insulin signalling drives glucose clearance into tissues at an accelerated rate, producing deceptively normal glucose values while concealing chronic metabolic dysfunction (37). This concealed state of hyperglycaemia is compounded by the inflammatory and mitogenic effects of sustained hyperinsulinaemia, which amplify systemic risk despite apparently reassuring glycaemic markers (34, 35, 38–41).

To overcome these diagnostic blind spots, alternative strategies are required. A more sensitive approach involves sequential monitoring of capillary glucose and ketone BHB over a minimum of seven consecutive evenings, either pre-dinner or at bedtime, and at least three hours post-prandially (16, 34, 35, 41, 42). This method directly assesses insulin’s suppressive effect on hepatic ketogenesis, providing an early marker of metabolic imbalance. In parallel, fasting venous blood biochemistry should incorporate markers that capture the endocrine, inflammatory, and vascular consequences of insulin excess, including plasminogen activator inhibitor-1, gamma-glutamyl transferase (GGT), leptin, homeostasis model assessment for insulin resistance (HOMA-IR), insulin-like growth factor-1 (IGF-1), vascular endothelial growth factor (VEGF), epidermal growth factor, and monocyte chemoattractant protein-1. Together, these indices provide a more comprehensive profile of the metabolic and vascular stress imposed by hyperinsulinaemia (16, 34, 35, 41).

Conventional standards of care (SOC) define normal glycaemia through fasting glucose and HbA1c values. However, reliance on these static markers fails to capture the metabolic consequences of persistent insulin excess (35, 37, 43). When laboratory results indicate glycaemia within the accepted reference interval yet clinical features such as atherosclerosis, or sleep apnoea are present, SCHI should be considered as a primary aetiological driver (44–54). These conditions share a unifying metabolic pattern in which euglycaemia is maintained at the cost of chronically elevated insulin, accompanied by suppressed ketogenesis (37, 53–55). Failure to recognise this compensatory glucose shunting leads to delayed diagnosis and intervention. The elevation of basal insulin, even within reference intervals, not only accelerates glucose uptake into cells but also produces false-negative results in standard clinical tests, thereby obscuring the detection of subclinical hyperglycaemia and early hyperinsulinaemia. This under-recognition allows metabolic damage to accumulate, such as chronic inflammatory signalling, impaired redox regulation, and endothelial dysfunction progress unchecked. Timely detection requires methods that assess both glucose and insulin dynamics, rather than static glucose endpoints to prevent misclassification and intervene before irreversible pathology develops.

This triad of euglycaemia, SCHI, and hypoketonaemia reflects a state of compensated metabolic dysregulation, in which chronic excess insulin exposure supresses the normal production of ketone bodies despite apparently stable glucose concentrations. Hypoketonaemia, therefore, provides a sensitive indicator of hidden metabolic imbalance, signifying insulin-mediated suppression of hepatic ketogenesis that may coexist with euglycaemia or overt hyperglycaemia. Recognition of this metabolic signature is critical, as the absence of abnormal fasting glucose or HbA1c does not equate to metabolic health when hyperinsulinaemia remains unaddressed.

Hypo-ketonaemia and *insulin-compensated euglycaemia* (ICE) define a hidden state of insulin and glycaemic pathology direction that is invisible to conventional standards. Insulin concentrations may fall within population reference intervals, but are elevated relative to individual metabolic capacity, thereby masking subclinical hyperglycaemia (fasting glucose ≤ 5.7 mmol/L due to insulin-compensation) and subclinical hyperinsulinaemia (≥ 8 μIU/mL). Hypoketonaemia-ICE is a metabolic state of hidden hyperglycaemia, in which glucose is cleared from the bloodstream, yet the total glycaemic load to the bloodstream is not captured. Where within reference range euglycaemia (fasting glucose ≤ 5.5 mmol/L) and HbA1c are maintained by compensatory insulin activity (hyperinsulinaemia) and revealed by chronic suppression of BHB (hypoketonaemia, < 0.5 mmol/L) most reliably detected through sequential daily evening capillary blood BHB measurement, where chronic hypoketonaemia demonstrates that basal insulin remains pathologically raised (17, 34, 35, 37, 38, 40, 43). A *personalised hyperinsulinaemia threshold* (PIT) is defined by chronic hypoketonaemia as a marker of insulin excess, which is individual-specific and not reliant on population reference ranges. Hypoketonaemia confirms SCHI even when glucose and serum insulin appear normal within standard reference ranges [37,38,43]. Hypoketonaemia-ICE captures the discordant state in which glycaemic indices appear unremarkable, yet underlying insulin-driven compensation reveals early metabolic disturbances.

A distinct metabolic state exists in which fasting glucose and HbA1c remain within conventional reference intervals, yet underlying hyperglycaemia is concealed by compensatory hyperinsulinaemia. In this condition, insulin levels may also appear ‘normal’ when interpreted against population-based ranges, but are pathologically elevated for the individual, driving excess glucose disposal and preventing hepatic ketogenesis. The most reliable indicator of this state is chronic evening hypoketonaemia, due to persistent insulin suppression of hepatic ketogenesis, defined as persistently low BHB (< 0.5 mmol/L) despite adequate fasting intervals (16, 34, 35, 40–42). This pattern reveals that basal insulin is set too high for the individual’s metabolic capacity, producing an artefactual appearance of normal glycaemia while masking early metabolic dysregulation or disease.

Insulin-Compensated Euglycaemia (ICE):

- Euglycaemia (normal fasting glucose and normal HbA1c) is achieved through insulin-compensation, not proper metabolic health.
- Detects “prediabetes,” which is stage 1 diabetes (38).
- Compensated by insulin, revealed by the clinical signature: hypoketonaemia.

Moving from population-based thresholds to individual metabolic capacity as the diagnostic anchor, the personalised hyperinsulinaemia threshold (PIT) refers to the detection of insulin excess relative to an individual’s metabolic capacity, rather than against population-derived reference intervals (Table 1). PIT is identified through the hypoketonaemia-ICE framework, where chronic evening hypoketonaemia (minimum 3 h post-prandial BHB < 0.5 mmol/L) reveals that basal insulin levels are elevated, even when fasting insulin remains within conventional laboratory ranges (17, 35). This approach recognises that insulin-induced mitochondrial, metabolic and endocrine dysregulation can occur below standard thresholds and provides a more sensitive and specific means of identifying early metabolic disease (16, 38, 40, 43, 56). Through the lens of hypoketonaemia-ICE, subclinical and clinical hyperinsulinaemia may be detected to within personalised thresholds, where the key determinant is not absolute serum insulin within population intervals, but the functional suppression of ketogenesis (hypoketonaemia).

**Table 1:** Conventional versus personalised hyperinsulinaemia threshold.

Feature	Conventional hyperinsulinaemia	Personalised hyperinsulinaemia threshold (PIT)
Definition	Insulin above population reference interval.	Insulin excess relative to an individual’s metabolic baseline, detected through functional markers.
Primary metric	Absolute serum insulin concentration or HOMA-IR	Chronic evening hypoketonaemia (suppressed beta-hydroxybutyrate) despite adequate 3 h post-prandial interval.
Reference standard	Laboratory-defined population ranges.	Individualised physiological thresholds (insulin–ketone relationship).
Sensitivity	Low, as many patients with early metabolic disease remain within “normal” insulin ranges.	High, as suppressed ketogenesis reveals insulin toxicity even with apparently normal glucose and insulin values.
Specificity	Limited, as elevated insulin may reflect transient or context-specific variation.	High, as persistent hypoketonaemia directly reflects sustained basal insulin excess.
Relation to glucose metrics	Detected only once hyperglycaemia emerges.	Detectable during euglycaemia, exposing hidden hypoketonaemia insulin-compensated euglycaemia (ICE).
Clinical utility	Identifies late-stage metabolic dysfunction.	Enables early detection and intervention before overt hyperglycaemia or changes in HbA1c.
Core limitation	Fails to recognise subclinical insulin toxicity in “normal” ranges.	Requires sequential ketone monitoring to reveal functional suppression.

Personalised hyperinsulinaemia threshold (PIT) reframes the detection of insulin excess by moving beyond population-based thresholds to individualised metabolic assessment. Through the framework of hypoketonaemia insulin-compensated euglycaemia (ICE), PIT is identified by the persistent suppression of hepatic ketogenesis (hypoketonaemia), most sensitively captured by evening beta-hydroxybutyrate measurement a 3-h post-prandial interval. Unlike conventional definitions of hyperinsulinaemia, which rely on absolute serum insulin concentrations or HOMA-IR, PIT identifies insulin toxicity even within apparently regular laboratory reference intervals, thereby providing greater sensitivity and specificity. This approach enables the recognition of early metabolic disease at a stage where fasting glucose and HbA1c remain deceptively normal, offering a more precise window for timely intervention.

We have previously published the effects of suppressing ketosis in the Ketosis Suppression and Ageing (KetoSAge) cohort, demonstrating changes in biomarkers associated with ageing and chronic disease, including insulin, HOMA-IR, BHB, leptin, IGF-1, thyroid hormone levels, and GGT (16, 34, 35, 39, 41). Building on this foundation, the present study focuses on female-specific endocrine responses, by examining the impact of long-term sustained nutritional ketosis (NK), euketonaemia (BHB ≥ 0.5–5 mmol/L), and the suppression of ketosis (SuK) and hypoketonaemia (BHB < 0.5 mmol/L), on key reproductive and hormonal biomarkers. Specifically, we investigated changes in circulating concentrations of SHBG, oestrogen, progesterone, testosterone, luteinising hormone (LH) and follicle-stimulating hormone (FSH), providing novel insights into the metabolic regulation of female hormonal health. SHBG may further refine the detection of SCHI and aid clinicians in determining risk and prognosis in chronic diseases.

## 2 Materials and methods

### 2.1 Participant characteristics and study design

**Figure 1:**
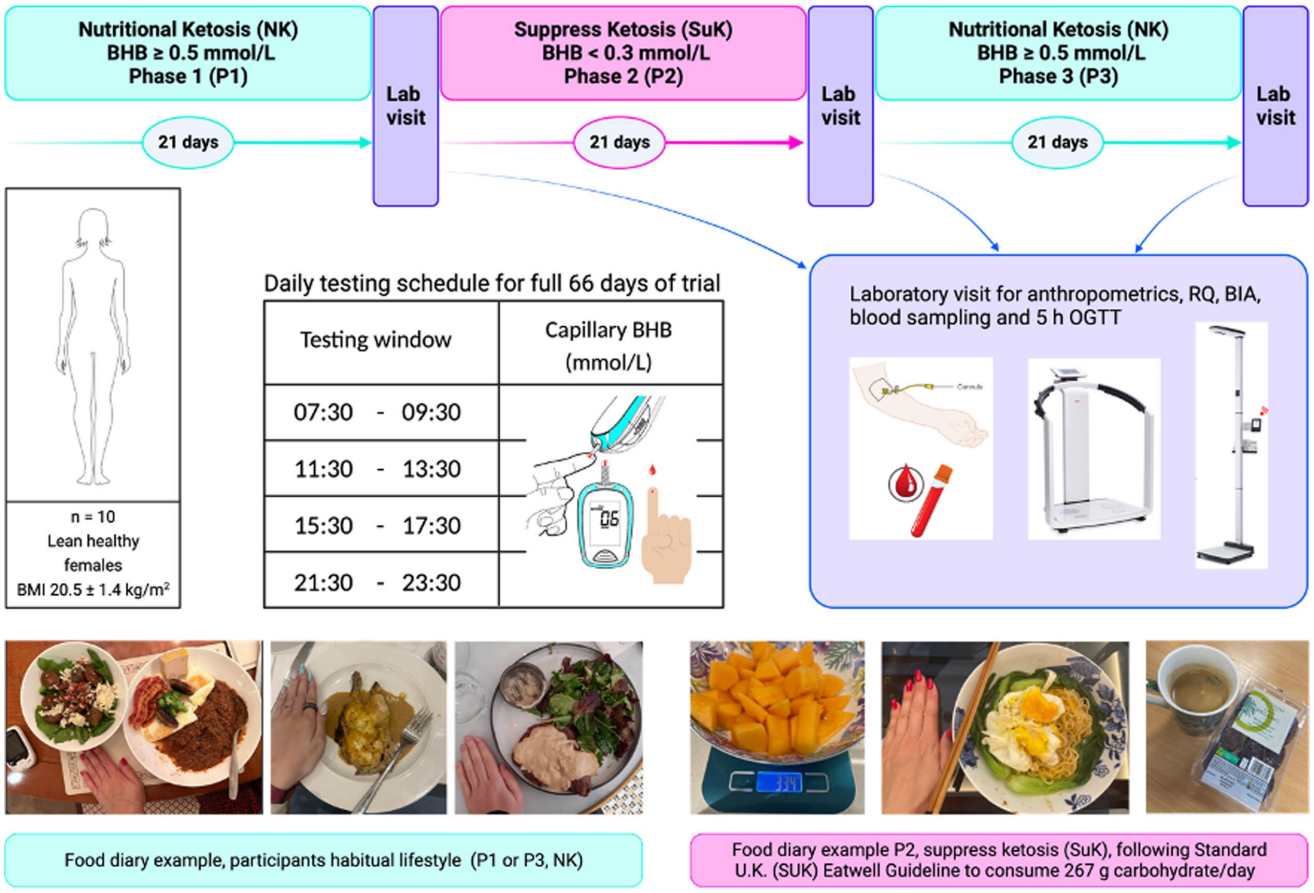
KetoSAge study design. Phases 1 and 3 covered the participants’ habitual nutritional ketosis lifestyle. Phase 2 was the interventional phase aimed at suppressing ketosis (SuK). Each phase was monitored via finger-prick testing of capillary beta-hydroxybutyrate (BHB) concentration (mmol/L). Testing was conducted four times per day, before to consuming any food, at evenly spaced intervals. At the end of each phase, participants underwent a laboratory testing day for body composition and biochemical tests. Participants were given an oral glucose tolerance test (75 g glucose in 250 mL water) as described in our earlier publication (35). Blood samples were taken at seven time points over 5 h. Whole-blood glucose and BHB were measured in real time using the Keto-Mojo™ meter, and a plasma insulin sensitivity assay was conducted later using an enzyme-linked immunosorbent assay (ELISA). Body mass index (BMI); oral glucose tolerance test (OGGT); and respiratory quotient (RQ). Reproduced from (16, 34), licensed under CC BY 4.0.

Ten lean (weight, 52.99 kg ± 4.24; height, 160.95 cm ± 7.28; BMI, 20.52 kg/m^2^ ± 1.39), healthy pre-menopausal women (age, 32.30 ± 8.97 years) who habitually followed a ketogenic diet participated in the KetoSAge study. This was an open-labelled, non-randomised crossover trial comprising three phases: baseline nutritional ketosis (NK; Phase 1, P1), suppression of ketosis (SuK; Phase 2, P2) and return to NK following removal of the intervention (Phase 3, P3) as previously described (16, 34, 35, 41). Participants self-reported adherence to a lifestyle maintaining NK for ≥ 6 months (mean 3.9 ± 2.3 years), providing sufficient time for metabolic adaptations. Baseline characteristics, adherence measures during experimental period, details on ketosis adaptation, and macronutrient composition with statistical analysis between phases, have been reported previously (16, 34, 35, 41). At the end of each study phase, participants attended the laboratory at 8:00 a.m. after a 12-h overnight fast for anthropometric measurements and blood sampling (Figure 1).

### 2.2 Anthropometric measurements

Upon arrival at the laboratory, height was measured (to the nearest 0.1 cm) using a stadiometer (Marsden HM-250P Leicester Height Measure). Body weight was measured (to the nearest 0.1 kg), and waist and hip circumference were obtained using a non-stretch anthropometric measuring tape (Seca® 201) while participants stood upright with feet together. The average of three measurements (cm) was used for analysis. Body mass index (BMI) and fat mass were measured by bioelectrical impedance (BIA) using Seca® (mBCA 514 Medical Body Composition Analyser, Gmbh&Co. KG, Hamburg, Germany). All measurements were taken following a 12-h overnight fast, with participants being with an empty bladder and wearing standardised light clothing (16, 34, 35).

### 2.3 Blood collection and measurement

As previously described, blood was drawn into ethylenediaminetetraacetic acid (EDTA) tubes (BD, Oxford, UK) before being centrifuged at 3,857 g for 10 min at 4 °C (Hettich Universal 320 R, Germany). Blood was also drawn into serum SST™ II Advance tubes with thrombin rapid clot activator and separation gel (BD, Oxford, UK) and left for 30 min at room temperature. Serum tubes were then centrifuged at 3,857 × g for 10 min at room temperature. Samples were either sent to SYNLAB Belgium (Alexander Fleming, 3–6,220 Heppignies–Company No: 0453.111.546) for analysis, or aliquoted into cryovial tubes under sterile conditions and stored at −80 °C for later analysis by Randox Ireland (55 Diamond Road, Crumlin, Co. Antrim, BT29 4QY, company number, NI015738), Clinilabs (London, UK) or in-house testing (16, 34, 35).

### 2.4 Blood marker analysis

Serum fasting insulin was measured using a Simple Plex Assay (Ella™, Bio-Techne, Minneapolis, USA). Fasted venous whole blood glucose and BHB concentrations were measured using the Keto-Mojo™ GKI multi-function meter (Keto-Mojo, Napa, CA, USA) (16, 34, 35, 57). The glucose ketone index (GKI) was calculated from whole blood readings obtained using a Keto-Mojo™ meter as follows: GKI = glucose (mmol/L) ÷ ketones (mmol/L). Leptin (DuoSet, R&D Systems, Minneapolis, MN, USA) and glucagon-like peptide (GLP-1; Abcam, Cambridge, UK) were quantified by ELISA from frozen serum samples, according to the manufacturer’s instructions. IGF-1, GGT, adiponectin, thyroid-stimulating hormone (TSH), free triiodothyronine (T3), reverse T3, thyroxine (T4) were measured externally by SYNALB, and serum iron was measured externally by Randox and Clinilabs as previously reported (16, 34, 35). Serum oestrogen, progesterone, testosterone, SHBG, LH, and FSH were measured by Clinilabs.

### 2.5 Statistical analysis

Data were checked for normality using the Shapiro–Wilk test. KetoSAge participants between the study phases (P1, P2, P3) were compared using either repeated measures (RM) one-way ANOVA with Tukey’s correction for multiple comparisons, or the Friedman test with Dunn’s correction for multiple comparisons, depending on the results of the normality tests. When the sphericity of data was not met, Geisser–Greenhouse corrections were also added. Graphed data are presented as mean ± SD. Data were analysed and graphed using GraphPad Prism (Version 10.6.1, GraphPad, United States).

Additionally, mixed effects models, sensitivity analyses, and their statistical tests were performed using R version 4.4.3. To account for our study’s design, linear mixed-effects models with a random intercept for each participant were used to compare the influence of different physiological variables on hormonal markers across the study phases. Sensitivity analyses were also conducted to account for variations in menstrual cycle stages. All models were implemented using the lmerTest:lmer function in R, with hypothesis testing based on Satterthwaite’s method for estimating the degrees of freedom and frequentist hypothesis testing. For the participant with hormone levels below the limit of detection, minimal detectable values for oestrogen, progesterone, and testosterone, were imputed. The results remained unchanged following this adjustment; therefore, statistical analyses on oestrogen, progesterone, and testosterone are presented with data of *n =* 9.

## 3 Results

### 3.1 Participant characteristics

Participants had a mean BMI of 20.52 (± 1.39 kg/m^2^) and a mean fat mass of 14.21 kg (± 2.55) at baseline (P1). Key findings from our analysis in KetoSAge participants are presented, as previously reported in KetoSAge studies (16, 34, 35, 41). Markers of BMI, fat mass, insulin, glucose, BHB, HOMA-IR, GKI, IGF-1, leptin, GLP-1, GGT, and free T3 were statistically significant from P1 to P2, and this trend reversed following P3. Markers of adiponectin, TSH, reverse T3, T4, and iron were not statistically significant. A summary of all markers investigated in this study across all phases is shown below (Table 2).

**Table 2:** BMI, fat mass, fasted insulin, glucose, BHB, HOMA-IR, GKI, leptin, IGF-1, GLP-1, adiponectin, iron, cortisol, TSH, free T3, reverse T3 and T4 across all phases in KetoSAge participants.

Biomarker	P1	P2	P3	*p*-value	P1 vs. P2	P2 vs. P3	P1 vs. P3
BMI (kg/m2)	20.52 (± 1.39)	21.54 (± 1.29)	20.82 (± 1.46)	<0.0001*	<0.0001*	<0.0001*	0.0734
Fat Mass (kg)	14.21 (± 2.55)	15.88 (± 2.23)	14.78 (± 2.20)	<0.0001*	<0.0001*	0.0018*	0.1102
Insulin (μIU/mL)	4.95 (± 1.24)	9.06 (± 2.13)	5.62 (± 1.83)	<0.0001*	<0.0001*	0.0001*	0.5686
Glucose (mmol/L)	4.23 (± 0.50)	5.01 (± 0.70)	4.24 (± 0.28)	0.0005*	0.0014*	0.0016*	0.9980
BHB (mmol/L)	2.43 (± 1.28)	0.18 (± 0.13)	2.31 (± 0.71)	0.0002*	0.0012*	<0.0001*	0.9638
HOMA-IR	0.93 (± 0.26)	2.03 (± 0.65)	1.07 (± 0.40)	<0.0001*	0.0010*	0.0024*	0.4719
GKI (Lab Day)	2.23 (± 1.20)	49.68 (± 42.62)	1.99 (± 0.60)	0.0001*	0.0024*	0.0024*	>0.9999
IGF-1 (μg/L)	149.30 (± 32.96)	273.40 (± 85.66)	136.90 (± 39.60)	0.0015*	0.0045*	0.0055*	0.4124
Leptin (ng/mL)	4.50 (± 3.66)	15.08 (± 8.00)	4.57 (± 3.48)	<0.0001*	0.0010*	0.0052*	>0.9999
GLP-1 (pg/mL)	1,383.18 (± 911.36)	576.72 (± 452.43)	1,471.85 (± 1,066.75)	0.0075*	0.0219*	0.0219*	>0.9999
GGT (U/L)	9.60 (±3.13)	12.40 (±2.55)	9.70 (±2.50)	0.0021*	0.0044*	0.0059*	0.9904
Adiponectin (μg/L)	9.08 (± 4.18)	10.75 (± 6.76)	8.70 (± 3.25)	0.4362	>0.9999	0.5391	>0.9999
TSH (mIU/L)	1.40 (± 0.74)	1.56 (± 0.75)	1.25 (± 0.81)	0.3146	0.7135	0.2833	0.7195
Free T3 (pmol/L)	3.82 (± 0.28)	5.50 (± 0.72)	4.05 (± 0.54)	<0.0001*	<0.0001*	0.0015*	0.2980
Reverse T3 (nmol/L)	0.29 (± 0.09)	0.26 (± 0.10)	0.25 (± 0.09)	0.5569	0.7181	0.9585	0.5498
T4 (pmol/L)	13.52 (± 1.61)	13.24 (± 1.49)	12.65 (± 0.66)	0.2120	0.8795	0.3168	0.2049
Iron (μmol/L)	16.62 (± 7.27)	14.40 (± 8.70)	11.76 (± 11.78)	0.1873	>0.9999	0.2209	0.3526

Beta-hydroxybutyrate (BHB); body mass index (BMI); free triiodothyronine (free T3); gamma-glutamyl transferase (GGT); glucose ketone index (GKI); glucagon-like peptide 1 (GLP-1); homeostasis model assessment for insulin resistance (HOMA-IR); insulin-like growth factor 1 (IGF-1); thyroxine (T4); thyroid stimulating hormone (TSH). Measurements were taken following each of the study phases: baseline nutritional ketosis (NK) P1; intervention to suppress ketosis (SuK) P2; and removal of SuK returning to NK P3. Samples were collected at 8 a.m. after a 12-h overnight fast. Data was analysed by RM one-way ANOVA or Friedman’s test (*n =* 10).

### 3.2 Suppression of ketosis is associated with changes in SHBG

**Figure 2:**
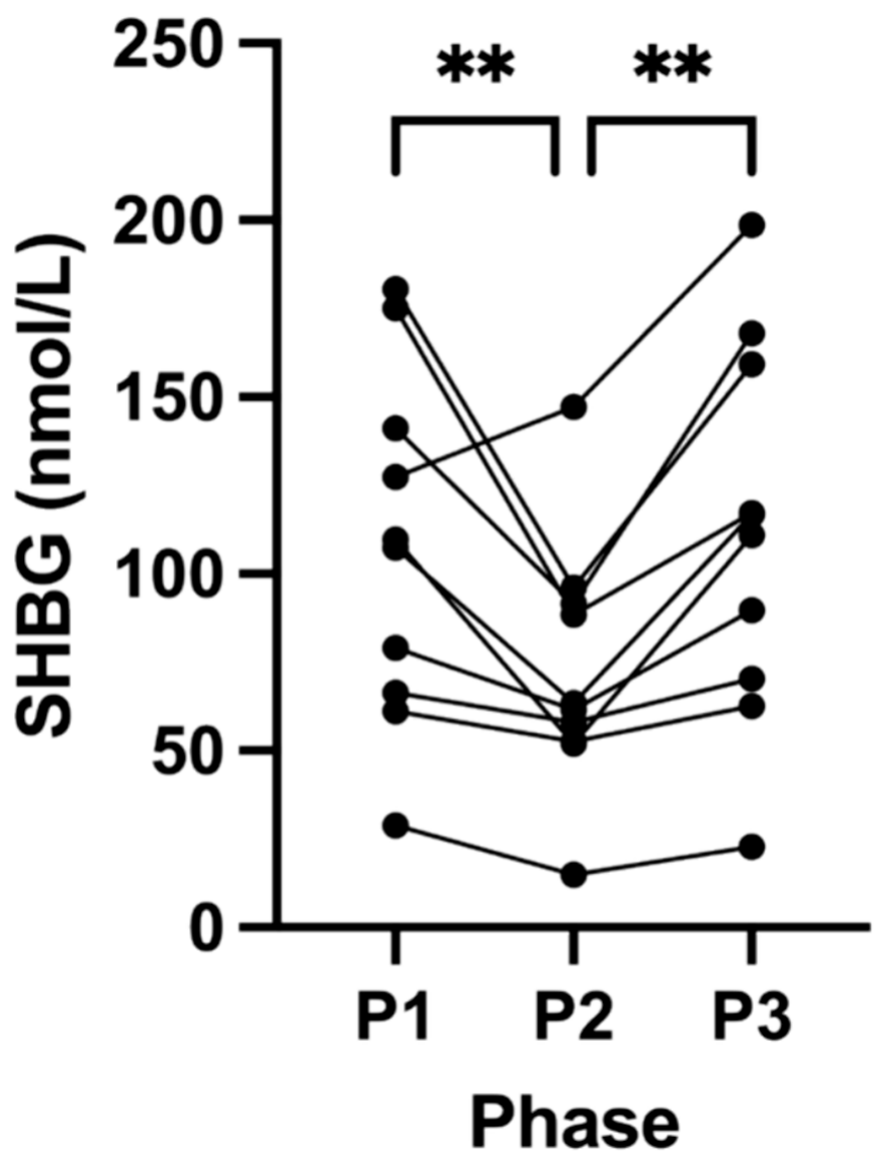
Levels of sex hormone-binding globulin (SHBG) across all phases in KetoSAge participants. Fasting serum concentrations of SHBG were measured following each of the study phases: baseline nutritional ketosis (NK), P1; intervention to suppress ketosis (SuK), P2; and removal of SuK returning to NK, P3. SHBG was measured by Clinilabs, London, UK. Samples were collected at 8 a.m. after a 12-h overnight fast (*n =* 10). Data were analysed by RM one-way ANOVA. **p < 0.01.

**Figure 3:**
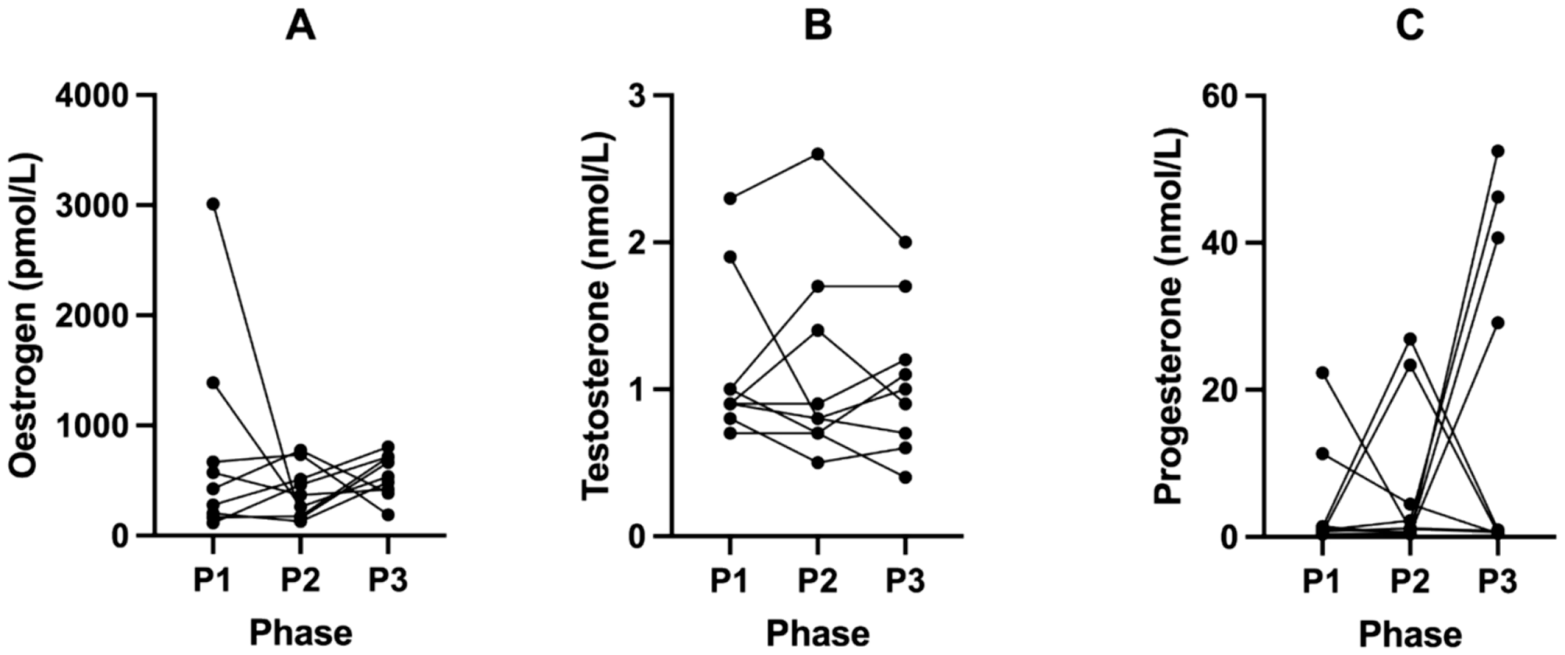
Levels of **(A)** Oestrogen, **(B)** Testosterone and **(C)** Progesterone across all phases in KetoSAge participants. Fasting serum concentrations of oestrogen, testosterone and progesterone were measured following each of the study phases: baseline nutritional ketosis (NK), P1; intervention to suppress ketosis (SuK), P2; and removal of SuK returning to NK, P3. Markers were measured by Clinilabs, London, UK. Samples were collected at 8 a.m. after a 12 h overnight fast (*n =* 9), as one participant’s hormone levels were below the limit of detection. Data were analysed by Friedman’s test.

**Figure 4:**
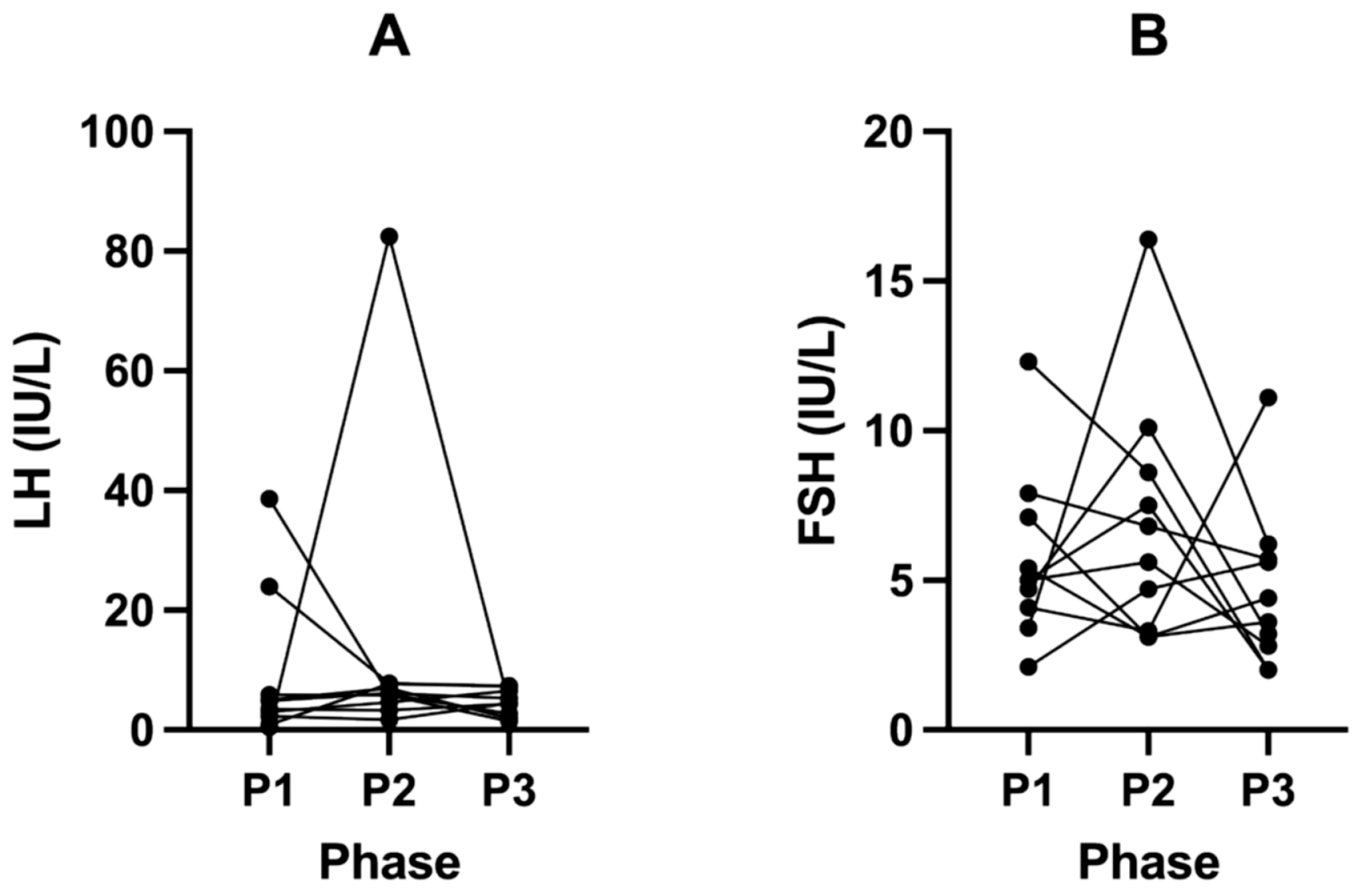
Levels of **(A)** Luteinising Hormone (LH) and **(B)** Follicle-Stimulating Hormone (FSH) across all phases in KetoSAge participants. Fasting serum concentrations of LH and FSH were measured following each of the study phases: baseline nutritional ketosis (NK), P1; intervention to suppress ketosis (SuK), P2; and removal of SuK returning to NK, P3. Markers were measured by Clinilabs, London, UK. Samples were collected at 8 a.m. after a 12-h overnight fast (*n =* 10). Data were analysed by Friedman’s test or RM one-way ANOVA.

Female hormone biomarkers (SHBG, oestrogen, testosterone, progesterone, LH and FSH) across all phases in KetoSAge participants are presented in Table 3. Following P2, SHBG significantly decreased from 107.70 nmol/L (± 49.74, P1) to 72.53 nmol/L (± 35.43, P2; *p* = 0.0060). This trend reversed following P3, where the SHBG significantly returned to participants’ baseline levels of 111.60 nmol/L (± 53.29, P3; *p* = 0.0025) compared to P2 (Table 3; Figure 2). Changes in oestrogen, testosterone, progesterone, LH and FSH were not statistically significant in this study (Table 3; Figures 3, 4).

**Table 3:** Female hormone biomarker panel in suppression of ketosis.

Biomarker	P1	P2	P3	*p*-value	P1 vs. P2	P2 vs. P3	P1 vs. P3
SHBG (nmol/L)	107.70 (± 49.74)	72.53 (± 35.43)	111.60 (± 53.29)	0.0015*	0.0060*	0.0025*	0.9146
Oestrogen (pmol/L)	758.30 (± 930.90)	397.10 (± 242.70)	545.80 (± 197.30)	0.6854	>0.9999	>0.9999	>0.9999
Testosterone (nmol/L)	1.16 (± 0.55)	1.12 (± 0.67)	1.07 (± 0.51)	0.8529	>0.9999	>0.9999	>0.9999
Progesterone (nmol/L)	4.45 (± 7.54)	6.77 (± 10.54)	19.10 (± 22.66)	0.9712	>0.9999	>0.9999	>0.9999
LH (IU/L)	8.84 (± 12.43)	13.23 (± 24.39)	4.57 (± 2.10)	0.8302	>0.9999	>0.9999	>0.9999
FSH (IU/L)	5.70 (± 2.85)	6.92 (± 4.11)	4.66 (± 2.72)	0.3799	0.7252	0.3481	0.7908

Follicle-stimulating hormone (FSH); luteinising hormone (LH); sex hormone binding globulin (SHBG). Measurements were taken following each of the study phases: baseline nutritional ketosis (NK) P1; intervention to suppress ketosis (SuK) P2; and removal of SuK returning to NK P3. Samples were collected at 8 a.m. after a 12-h overnight fast. Data was analysed by RM one-way ANOVA or Friedman’s test (*n =* 10; SHBG, LH, FSH, and *n =* 9; oestrogen, progesterone, testosterone).

### 3.3 Relationship of female hormone changes with basal insulin

**Figure 5:**
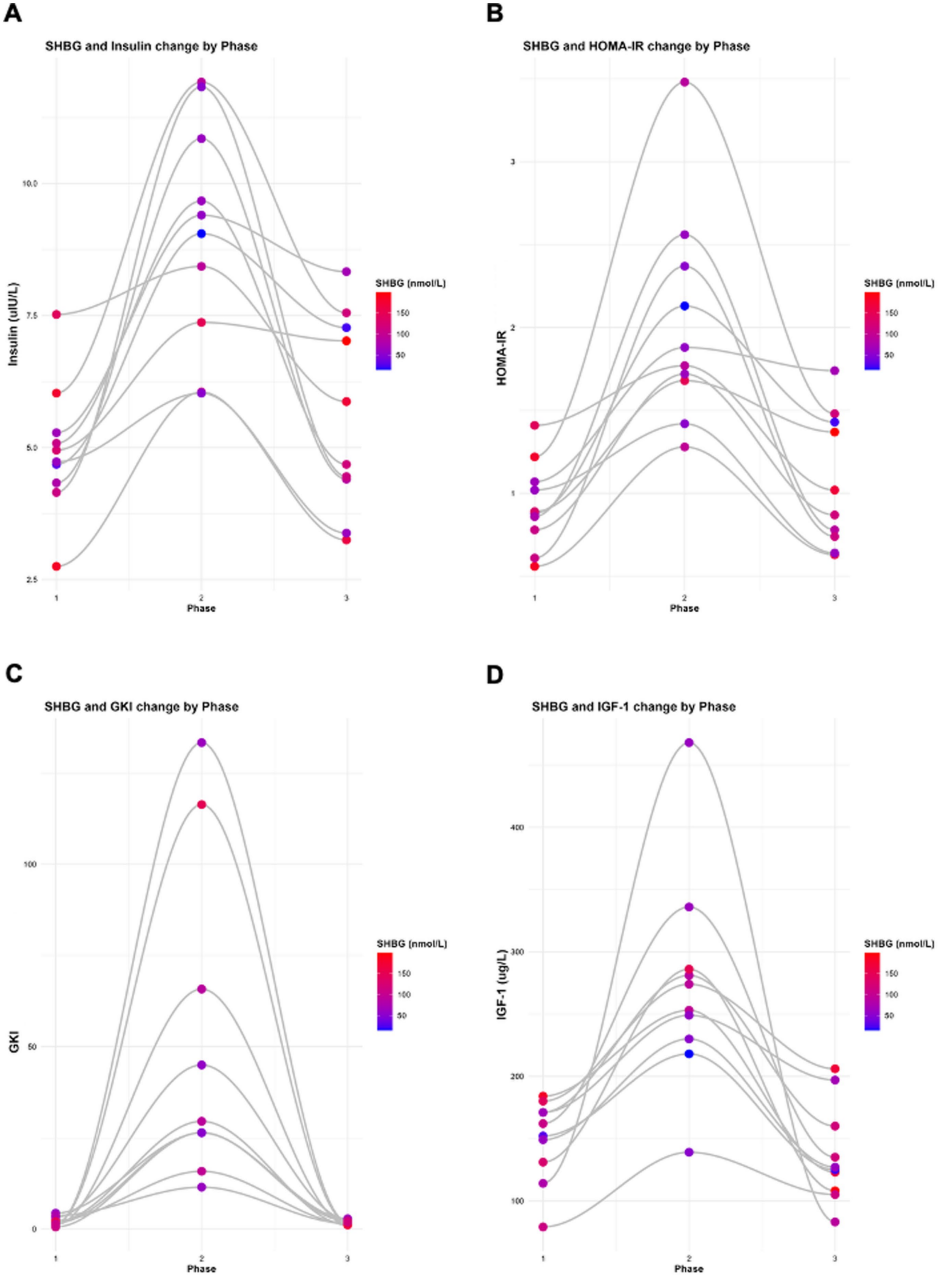
Changes in SHBG with **(A)** insulin, **(B)** HOMA-IR, **(C)** GKI, and **(D)** IGF-1 across all study phases. Analyses were performed and graphs were generated using RStudio with ggplot2.

Data presented in Table 4 demonstrate the relationship between female hormonal markers (SHBG, oestrogen, testosterone, progesterone, LH and FSH) and changes in insulin across the three phases and accounting for individual variability among the KetoSAge participants. A significant inverse association was observed between insulin and SHBG [effect estimate (*β*) = −7.5030, *p* = 0.0010; Table 4A; Figure 5]. In contrast, the relationships between insulin and oestrogen (*β* = −40.2700, *p* = 0.3380), testosterone (*β* = −0.0183, *p* = 0.4800), progesterone (*β* = −0.1788, *p* = 0.8810), LH (*β* = 0.6588, *p* = 0.5800), and FSH (*β* = 0.1620, *p* = 0.5181) were not statistically significant (Table 4A).

**Table 4:** Change in biomarkers SHBG, oestrogen, testosterone, progesterone, LH and FSH with changes in insulin across all phases in KetoSAge participants.

(A)		
Model	Effect estimate	*p-*value
SHBG ~ Insulin	−7.5030	0.0010*
Oestrogen ~ Insulin	−40.2700	0.3380
Testosterone ~ Insulin	−0.0183	0.4800
Progesterone ~ Insulin	−0.1788	0.8810
LH ~ Insulin	0.6588	0.5800
FSH ~ Insulin	0.1620	0.5181

(B) Log transformed		
Model	Effect estimate	*p-*value
SHBG ~ Insulin	−0.6155	7.85E-06*
Oestrogen ~ Insulin	−0.2505	0.5290
Testosterone ~ Insulin	−0.1525	0.3290
Progesterone ~ Insulin	−0.2915	0.7330
LH ~ Insulin	0.4197	0.3970
FSH ~ Insulin	0.2793	0.2848

Measurements were taken following each of the study phases: baseline nutritional ketosis (NK), P1; intervention suppress ketosis (SuK), P2; and removal of SuK returning to NK, P3. Samples were collected at 8 a.m. after a 12 h overnight fast (*n =* 10; SHBG, LH, FSH, and *n =* 9; oestrogen, progesterone, testosterone).

**Figure 6:**
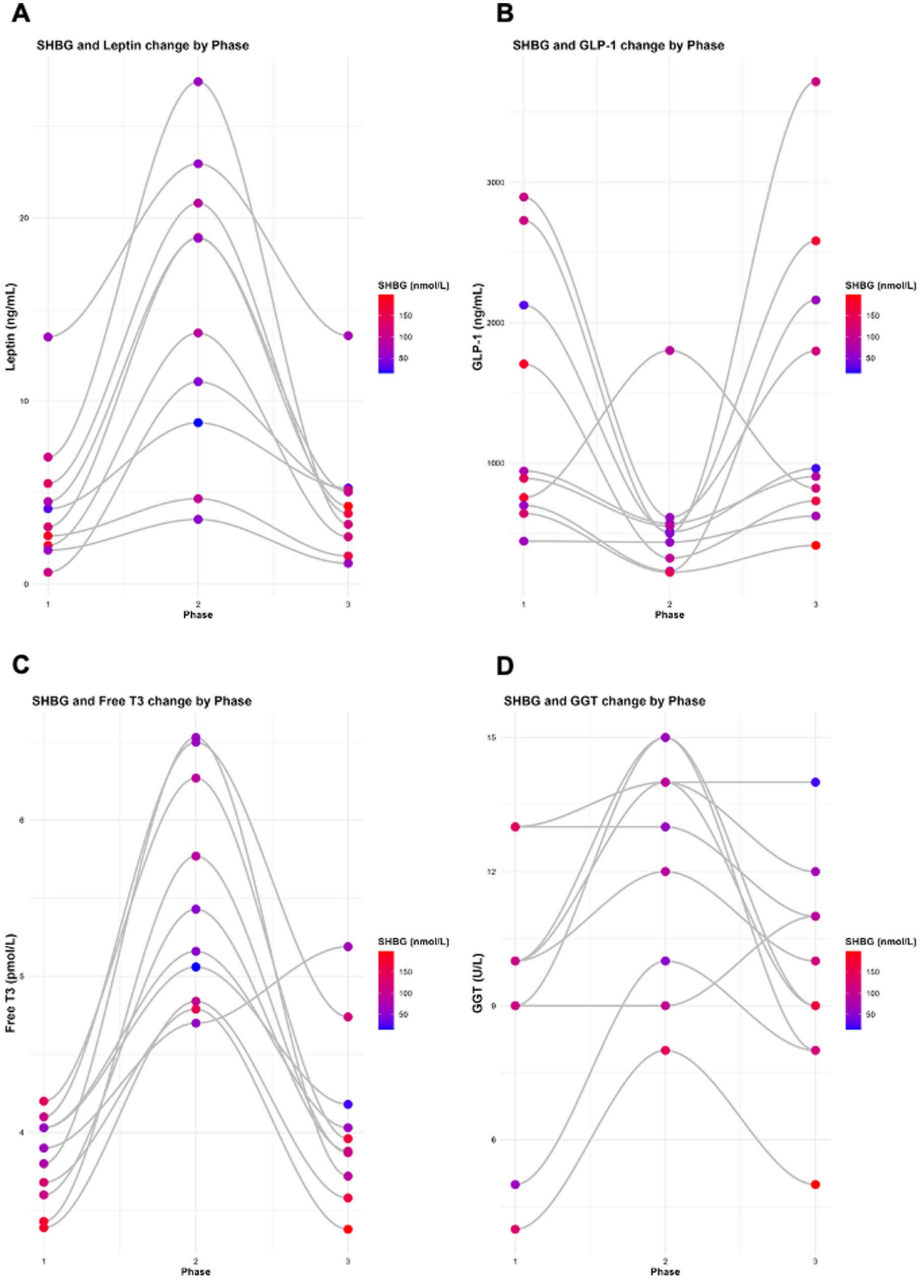
Changest in SHBG with **(A)** leptin, **(B)** GLP-1, **(C)** Free T3, and **(D)** GGT) across all study phases. Analysis were performed and graphs were generated using RStudio with ggplot2.

When data were log-transformed to account for the scale differences across variables, the observed relationships remained consistent. A significant inverse association was observed between insulin and SHBG (*β* = −0.6155, *p* = 7.85 × 10^−6^; Table 4B; Figure 6). In contrast, the relationships between insulin and oestrogen (*β* = −0.2505, *p* = 0.5290), testosterone (*β* = −0.1525, *p* = 0.3290), progesterone (*β* = −0.2915, *p* = 0.7330), LH (*β* = 0.4197, *p* = 0.3970), and FSH (*β* = 0.2793, *p* = 0.2848) were not statistically significant (Table 4B). In both raw data and log-transformed data, SHBG shows a significant inverse association with insulin.

### 3.4 Relationship of sex hormone changes with HOMA-IR

Table 5 shows the relationship between key sex hormone markers (SHBG, oestradiol, testosterone, progesterone, LH, and FSH) and dynamic changes in HOMA-IR across the three intervention phases. These correlations reflect inter-individual variability among KetoSAge participants and capture temporal endocrine adaptations. Similarly, a significant inverse association was observed between HOMA-IR and SHBG (*β* = −27.4030, *p* = 0.0012; Table 5A; Figure 5). In contrast, the relationships between HOMA-IR and oestrogen (*β* = −169.9000, *p* = 0.2790), testosterone (*β* = −0.0622, *p* = 0.5150), progesterone (*β* = −0.0323, *p* = 0.9940), LH (*β* = 2.9110, *p* = 0.5140), and FSH (*β* = 0.6227, *p* = 0.5076) were not statistically significant (Table 5A).

**Table 5:** Change in biomarkers SHBG, oestrogen, testosterone, progesterone, LH and FSH with changes in HOMA-IR across all phases in KetoSAge participants.

(A)		
Model	Effect estimate	*p*-value
SHBG ~ HOMA-IR	−27.4030	0.0012*
Oestrogen ~ HOMA-IR	−169.9000	0.2790
Testosterone ~ HOMA-IR	−0.0622	0.5150
Progesterone ~ HOMA-IR	−0.0323	0.9940
LH ~ HOMA-IR	2.9110	0.5140
FSH ~ HOMA-IR	0.6227	0.5076

(B) Log-transformed		
Model	Effect estimate	*p*-value
SHBG ~ HOMA-IR	−0.4898	7.62E-06*
Oestrogen ~ HOMA-IR	−0.3204	0.3270
Testosterone ~ HOMA-IR	−0.1268	0.3070
Progesterone ~ HOMA-IR	−0.2444	0.7290
LH ~ HOMA-IR	0.2440	0.5520
FSH ~ HOMA-IR	0.2372	0.2720

When data were log-transformed to account for the scale differences across variables, the observed relationships remained consistent. A significant inverse association was observed between HOMA-IR and SHBG (*β* = −0.4898, *p* = 7.62 × 10^−6^; Table 5B; Figure 6). In contrast, the relationships between HOMA-IR and oestrogen (*β* = −0.3204, *p* = 0.3270), testosterone (*β* = −0.1268, *p* = 0.3070), progesterone (*β* = −0.2444, *p* = 0.7290), LH (*β* = 0.2440, *p* = 0.5520), and FSH (*β* = 0.2372, *p* = 0.2720) were not statistically significant (Table 5B). In both raw data and log-transformed data, SHBG shows a significant inverse association with HOMA-IR.

### 3.5 Relationship of sex hormone changes with GKI

Data presented in Table 6 show the relationship between female hormone markers (SHBG, oestrogen, testosterone, progesterone, LH and FSH) and changes in GKI across the three phases, accounting for individual variability among the KetoSAge participants. Similarly, a significant inverse association was observed between GKI and SHBG (*β* = −0.4102, *p* = 0.0183; Table 6A; Figure 5C). In contrast, the relationships between GKI and oestrogen (*β* = −1.8420, *p* = 0.5680), testosterone (*β* = −0.0009, *p* = 0.6520), progesterone (*β* = −0.0862, *p* = 0.3397), LH (*β* = 0.0103, *p* = 0.9099), and FSH (*β* = 0.0132, *p* = 0.4890) were not statistically significant (Table 6A).

**Table 6:** Change in biomarkers SHBG, oestrogen, testosterone, progesterone, LH and FSH with changes in GKI across all phases in KetoSAge participants.

(A)		
Model	Effect estimate	*p*-value
SHBG ~ GKI	−0.4102	0.0183*
Oestrogen ~ GKI	−1.8420	0.5680
Testosterone ~ GKI	−0.0009	0.6520
Progesterone ~ GKI	−0.0862	0.3397
LH ~ GKI	0.0103	0.9099
FSH ~ GKI	0.0132	0.4890

(B) Log-transformed		
Model	Effect estimate	*p*-value
SHBG ~ GKI	−0.1282	1.50E-05*
Oestrogen ~ GKI	−0.1336	0.1790
Testosterone ~ GKI	−0.0290	0.3980
Progesterone ~ GKI	−0.1422	0.5073
LH ~ GKI	0.1072	0.3900
FSH ~ GKI	0.0750	0.2530

**Figure 7:**
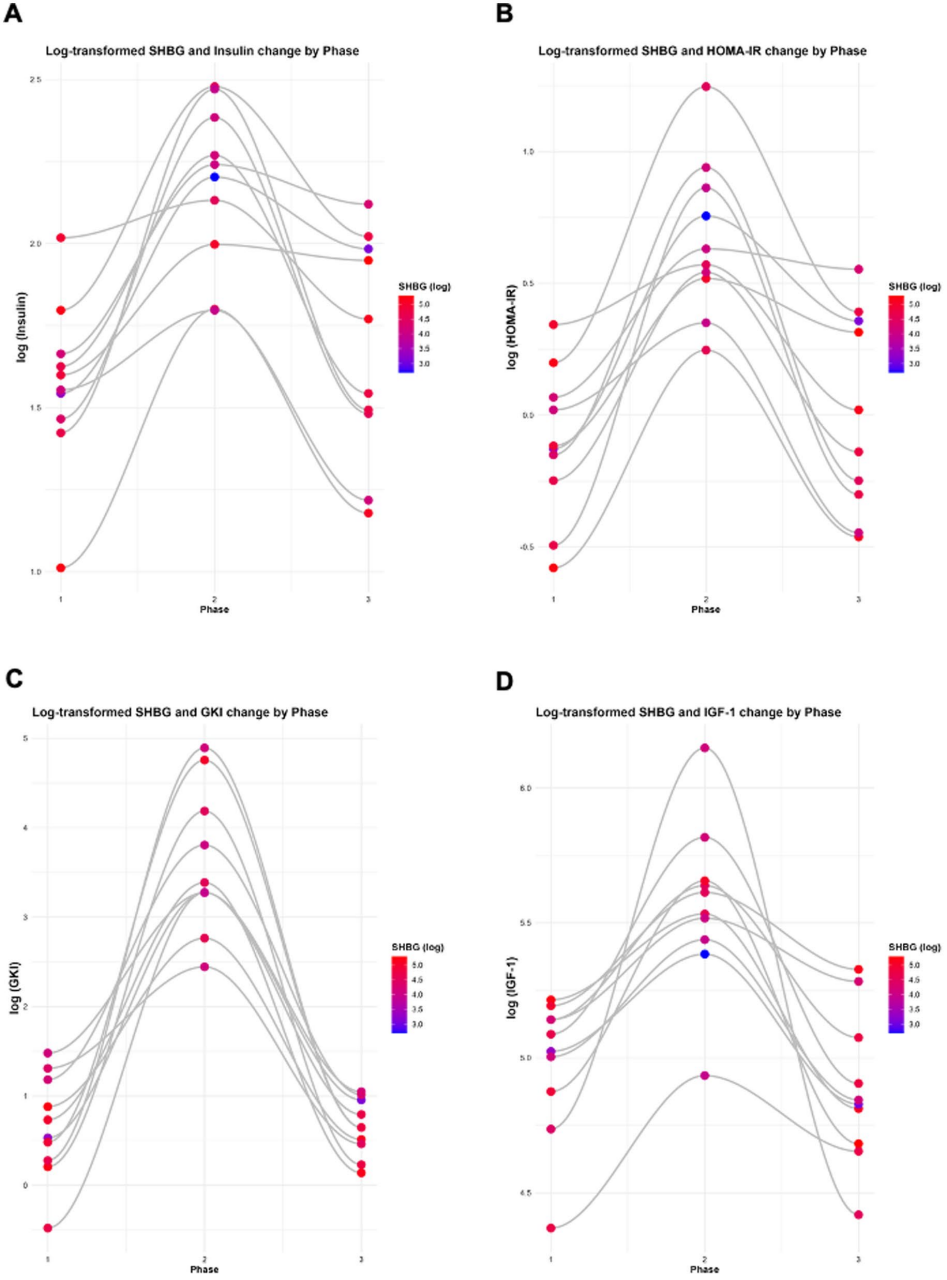
Log-transformed changes in SHBG with **(A)** insulin, **(B)** HOMA-IR, **(C)** GKI, and **(D)** IGF-1 across all study phases. Analyses were performed and graphswere generated using RStudio with ggplot2.

When data were log-transformed to account for the scale differences across variables, the observed relationships remained consistent. A significant inverse association was observed between GKI and SHBG (*β* = −0.1282, *p* = 1.50 × 10^−5^; Table 6B; Figure 7C). In contrast, the relationships between GKI and oestrogen (*β* = −0.1336, *p* = 0.1790), testosterone (*β* = −0.0290, *p* = 0.3980), progesterone (*β* = −0.1422, *p* = 0.5073), LH (*β* = 0.1072, *p* = 0.3900), and FSH (*β* = 0.0750, *p* = 0.2530) were not statistically significant (Table 6B). In both raw data and log-transformed data, SHBG shows a significant inverse association with GKI.

### 3.6 Relationship of sex hormone changes with leptin

Data presented in Table 7 demonstrate the relationship between female hormone markers (SHBG, oestrogen, testosterone, progesterone, LH and FSH) and changes in leptin across the three phases, accounting for individual variability among the KetoSAge participants. Similarly, a significant inverse association was observed between leptin and SHBG (*β* = −2.6110, *p* = 0.0016; Table 7A; Figure 6A). In contrast, the relationships between leptin and oestrogen (*β* = −8.6700, *p* = 0.5513), testosterone (*β* = −0.0057, *p* = 0.5420), progesterone (*β* = −0.0586, *p* = 0.8863), LH (*β* = 0.1138, *p* = 0.7812), and FSH (*β* = 0.0624, *p* = 0.4680) were not statistically significant (Table 7A).

**Table 7:** Change in biomarkers SHBG, oestrogen, testosterone, progesterone, LH and FSH with changes in leptin across all phases in KetoSAge participants.

(A)		
Model	Effect estimate	*p*-value
SHBG ~ Leptin	−2.6110	0.0016*
Oestrogen ~ Leptin	−8.6700	0.5513
Testosterone ~ Leptin	−0.0057	0.5420
Progesterone ~ Leptin	−0.0586	0.8863
LH ~ Leptin	0.1138	0.7812
FSH ~ Leptin	0.0624	0.4680

(B) Log-transformed		
Model	Effect estimate	*p*-value
SHBG ~ Leptin	−0.2426	0.0001*
Oestrogen ~ Leptin	−0.1045	0.5210
Testosterone ~ Leptin	−0.0973	0.1420
Progesterone ~ Leptin	0.1841	0.5970
LH ~ Leptin	0.1229	0.5460
FSH ~ Leptin	0.0898	0.4030

Measurements were taken following each of the study phases: baseline nutritional ketosis (NK), P1; intervention suppress ketosis (SuK), P2; and removal of SuK returning to NK, P3. Samples were collected at 8 a.m. after a 12-h overnight fast (*n =* 10; SHBG, LH, FSH, and *n =* 9; oestrogen, progesterone, testosterone).

**Figure 8:**
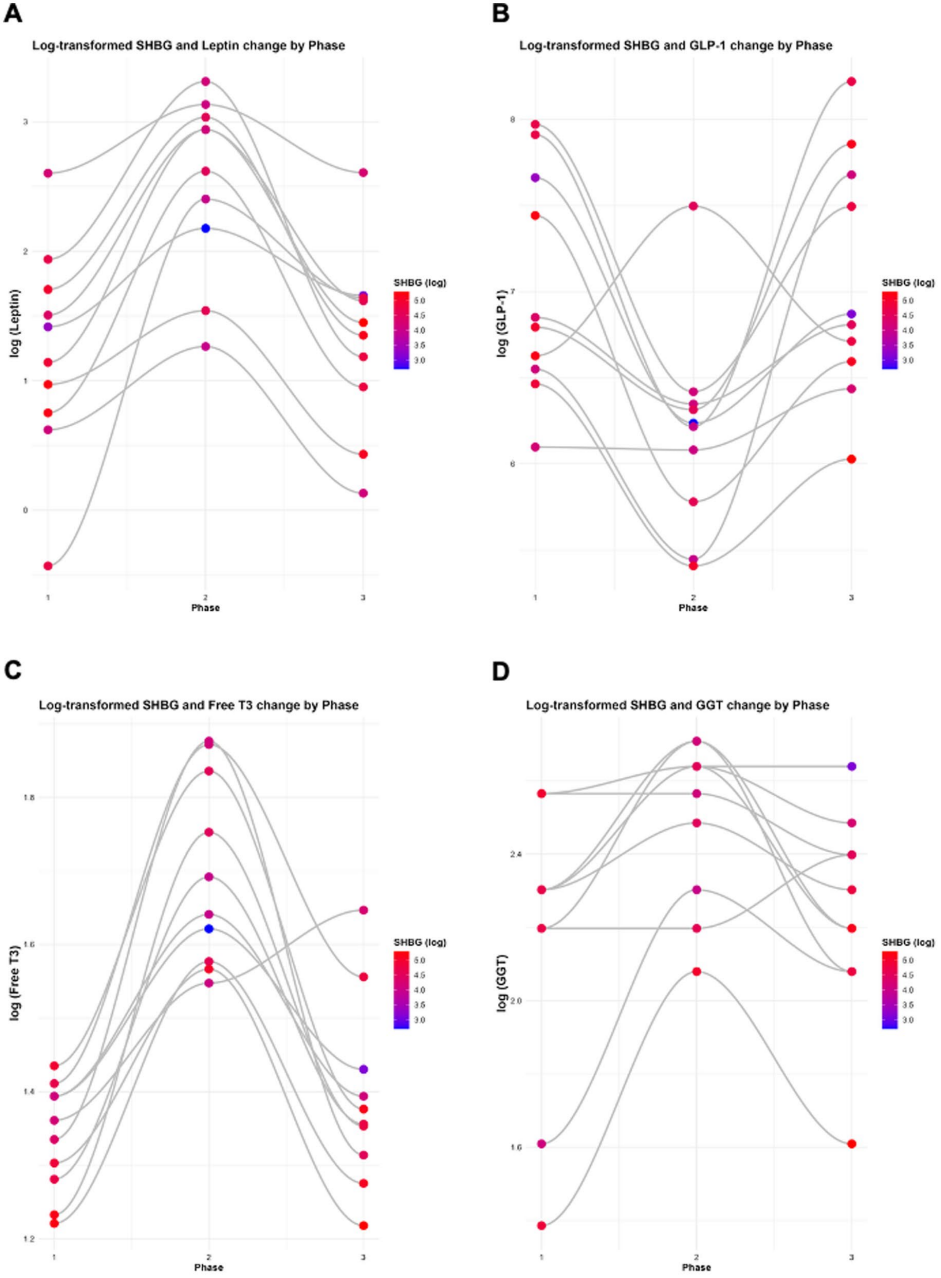
Log-transformed changes in SHBG with **(A)** leptin, **(B)** GLP-1, **(C)** free T3, and **(D)** GGT across all study phases. Analyses were performed and graphs were generated using RStudio with ggplot2.

When data were log-transformed to account for the scale differences across variables, the observed relationships remained consistent. A significant inverse association was observed between leptin and SHBG (*β* = −0.2426, *p* = 0.0001; Table 7B; Figure 8A). In contrast, the relationships between leptin and oestrogen (*β* = −0.1045, *p* = 0.5210), testosterone (*β* = −0.0973, *p* = 0.1420), progesterone (*β* = 0.1841, *p* = 0.5970), LH (*β* = 0.1229, *p* = 0.5460), and FSH (*β* = 0.0898, *p* = 0.4030) were not statistically significant (Table 7B). In both raw data and log-transformed data, SHBG shows a significant inverse association with leptin.

### 3.7 Relationship of SHBG changes with metabolic parameters

Table 8 shows the relationship between SHBG and changes in metabolic markers (insulin, HOMA-IR, GKI, leptin, IGF-1, GLP-1, adiponectin, TSH, free T3, reverse T3, and GGT) across the three phases and accounting for individual variability among the KetoSAge participants. As previously reported above, significant inverse associations were observed between SHBG and insulin, HOMA-IR, GKI, and leptin (Figures 5, 6). Further analyses, including other biomarkers tested, were conducted to assess the relationship between SHBG and the remaining metabolic biomarkers. Statistically significant associations were found between SHBG and IGF-1 (*β* = −0.1624, *p* = 0.0172), GLP-1 (*β* = 0.0152, *p* = 0.0295), free T3 (*β* = −22.4130, p = 0.0001), and GGT (*β* = −7.7060, *p* = 0.0024; Table 8A; Figures 5, 6). Adiponectin, TSH, and reverse T3 were not statistically significant (Table 8A).

**Table 8:** Change in SHBG with changes in insulin, HOMA-IR, GKI, leptin, IGF-1, GLP-1, adiponectin, TSH, free T3, reverse T3, and GGT, across all phases in KetoSAge participants.

(A)		
Model	Effect estimate	*p*-value
SHBG ~ Insulin	−7.5030	0.0010*
SHBG ~ HOMA-IR	−27.4030	0.0012*
SHBG ~ GKI	−0.4102	0.0183*
SHBG ~ Leptin	−2.6110	0.0016*
SHBG ~ IGF-1	−0.1624	0.0172*
SHBG ~ GLP-1	0.0152	0.0295*
SHBG ~ Adiponectin	−2.0150	0.2550
SHBG ~ TSH	−21.9100	0.0723
SHBG ~ Free T3	−22.4130	0.0001*
SHBG ~ Reverse T3	−8.1370	0.9148
SHBG ~ GGT	−7.7060	0.0024*

(B) Log transformed		
Model	Effect estimate	*p*-value
SHBG ~ Insulin	−0.6155	7.85E-06*
SHBG ~ HOMA-IR	−0.4898	7.62E-06*
SHBG ~ GKI	−0.1282	1.50E-05*
SHBG ~ Leptin	−0.2426	0.0001*
SHBG ~ IGF-1	−0.4163	0.0019*
SHBG ~ GLP-1	0.2477	0.0015*
SHBG ~ Adiponectin	−0.1651	0.4290
SHBG ~ TSH	−0.1951	0.0899
SHBG ~ Free T3	−1.1155	3.64E-06*
SHBG ~ Reverse T3	−0.0457	0.8110
SHBG ~ GGT	−0.6835	0.0058*

Measurements were taken following each of the study phases: baseline nutritional ketosis (NK), P1; intervention suppress ketosis (SuK), P2; and removal of SuK returning to NK, P3. Samples were collected at 8 a.m. after a 12-h overnight fast; (*n =* 10).

When data were log-transformed to account for the scale differences across variables, the observed relationships remained consistent. As previously reported, significant inverse associations were observed between log-transformed SHBG and insulin, HOMA-IR, GKI, and leptin (Figures 7, 8). Statistically significant associations were also found between SHBG and IGF-1 (*β* = −0.4163, *p* = 0.0019), GLP-1 (*β* = 0.2477, *p* = 0.0015), free T3 (*β* = −1.1155, *p* = 3.64 × 10^−6^), and GGT (*β* = −0.6835, *p* = 0.0058; Table 8B; Figures 7, 8). Adiponectin, TSH and reverse T3 were not statistically significant (Table 8B).

### 3.8 Interaction between sex hormones and the menstrual cycle

To investigate the interaction between female sex hormones and menstrual cycle, an interaction model was constructed including sex hormones, study phase, and menstrual cycle (Table 9). Both raw and log-transformed data for female sex hormones, study phase and stage of the menstrual cycle were analysed. The menstrual cycle stage was not found to be an independent and significantly interacting predictor of female hormone changes in this study.

**Table 9:** Changes in SHBG, oestrogen, testosterone, progesterone, LH and FSH accounting for study phase and menstrual cycle in KetoSAge participants.

(A)		
Model	Effect estimate	*p*-value
SHBG ~ Phase * Cycle Stage	Phase: Luteal: 0.4911 Phase: Ovulation: 25.5755	Phase: Luteal: 0.9816 Phase: Ovulation: 0.6382
Oestrogen ~ Phase * Cycle Stage	Phase: Luteal: −117.8300 Phase: Ovulation: 264.9500	Phase: Luteal: 0.6730 Phase: Ovulation: 0.7230
Testosterone ~ Phase * Cycle Stage	Phase: Luteal: 0.0219 Phase: Ovulation: −0.2524	Phase: Luteal: 0.9186 Phase: Ovulation: 0.6412
Progesterone ~ Phase * Cycle Stage	Phase: Luteal: 7.7710 Phase: Ovulation: −3.5290	Phase: Luteal: 0.1980 Phase: Ovulation: 0.8240
LH ~ Phase * Cycle Stage	Phase: Luteal: 2.0620 Phase: Ovulation: −34.8570	Phase: Luteal: 0.7411 Phase: Ovulation: 0.0504
FSH ~ Phase * Cycle Stage	Phase: Luteal: 0.9143 Phase: Ovulation: −8.6900	Phase: Luteal: 0.4250 Phase: Ovulation: 0.0097*

(B) Log-transformed		
Model	Effect estimate	*p*-value
SHBG ~ Phase * Cycle Stage	Phase: Luteal: 0.0577 Phase: Ovulation: 0.6489	Phase: Luteal: 0.7870 Phase: Ovulation: 0.2360
Oestrogen ~ Phase * Cycle Stage	Phase: Luteal: −0.0080 Phase: Ovulation: 0.4971	Phase: Luteal: 0.9840 Phase: Ovulation: 0.6380
Testosterone ~ Phase * Cycle Stage	Phase: Luteal: 0.1181 Phase: Ovulation: 0.0049	Phase: Luteal: 0.5250 Phase: Ovulation: 0.9920
Progesterone ~ Phase * Cycle Stage	Phase: Luteal: 0.1135 Phase: Ovulation: −0.5425	Phase: Luteal: 0.8630 Phase: Ovulation: 0.7590
LH ~ Phase * Cycle Stage	Phase: Luteal: 0.4450 Phase: Ovulation: −1.0549	Phase: Luteal: 0.3245 Phase: Ovulation: 0.3939
FSH ~ Phase * Cycle Stage	Phase: Luteal: 0.1724 Phase: Ovulation: −1.0718	Phase: Luteal: 0.3972 Phase: Ovulation: 0.0629

Measurements were taken following each of the study phases: baseline nutritional ketosis (NK), P1; intervention suppress ketosis (SuK), P2; and removal of SuK returning to NK, P3. Samples were collected at 8 a.m. after a 12-h overnight fast; (*n =* 10).

When analysed relative to the follicular phase, the menstrual cycle stage was a significant predictor of FSH levels (Phase: Luteal, *β* = 0.9143 and Phase: Ovulation, *β* = −8.6900; Phase: Luteal, *p* = 0.4250, and Phase: Ovulation, *p* = 0.0097) (Table 9A). However, this significant association became marginal when the data were log-transformed. In the log-transformed model, the menstrual cycle stage was not a significant independent or interacting predictor for any of the female hormone biomarkers assessed (Table 9B).

## 4 Discussion

The female reproductive system is closely regulated by nutritional status and overall energy balance. Metabolic conditions, such as hyperinsulinaemia and insulin resistance can profoundly impact female hormone levels and reproductive function, particularly in conditions like PCOS (4, 59, 60, 68). Despite growing interest, the complex pathways by which metabolic health and nutritional signals influence reproductive function remain incompletely understood. Recent studies have investigated the potential role of SHBG, a hepatic glycoprotein, in metabolic dysfunction, due to its inverse relationship with hyperinsulinaemia, insulin resistance and hepatic fat accumulation (58–60). Lower circulating SHBG levels have been correlated with markers of metabolic dysregulation, suggesting its possible utility as a biomarker for hyperinsulinaemia, insulin resistance and metabolic dysfunction-associated steatosis liver disease (MASLD). The present study examined the dynamics of key female hormone biomarkers across three menstrual cycle phases in a cohort of healthy premenopausal women who followed a ketogenic lifestyle with sustained NK (mean duration: 3.9 ± 2.3 years). A more precise understanding of how metabolic state influences reproductive hormone regulation is crucial to advancing women’s health across the lifespan.

We observed a significant decrease in SHBG levels following suppression of ketosis (P2, hypoketonaemia for 21 days), with a subsequent marked return to baseline values upon re-establishment of NK (P3, euketonaemia). Linear mixed-effects modelling further revealed strong associations between SHBG and several metabolic and hormonal markers, including insulin, HOMA-IR, GKI, leptin, IGF-1, GLP-1, free T3 and GGT. These findings are consistent with the role of SHBG as a marker responsive to both insulin signalling and hepatic function (15, 23, 60–67), two systems known to be modulated by long-term ketosis, euketonaemia (16, 67, 68). Observational studies have consistently shown an inverse relationship between SHBG and insulin, with lower SHBG concentrations linked to higher insulin levels and an increased risk of T2DM, independent of circulating sex steroid levels in both men and women (66). This inverse relationship is particularly relevant in the context of insulin resistance and hepatic dysfunction, both of which are central features of MetS and are ameliorated through carbohydrate restriction. The return of SHBG to baseline levels upon reintroduction of ketosis further supports the metabolic responsiveness of SHBG and highlights its potential utility as a dynamic biomarker of metabolic-endocrine status in women.

The liver plays a central role in the regulation of systemic insulin sensitivity and sex steroid bioavailability, primarily through its modulation of SHBG synthesis in response to insulin signalling (65, 69). Notably, women with both low SHBG and elevated hepatic fat content have been shown to exhibit the highest insulin concentrations (70). This inverse relationship between SHBG and insulin is particularly pronounced among women with greater liver fat accumulation (62, 70), suggesting a compounded metabolic risk in the presence of both hepatic steatosis and decreased SHBG. These findings highlight the liver’s central role in integrating metabolic and reproductive functions, a relationship that becomes increasingly relevant in midlife women as oestrogen levels begin to decline. Consistent with this, lower endogenous SHBG levels have been robustly associated with increased risk for cardiometabolic disorders and non-alcoholic fatty liver disease across both sexes and age groups (24, 71, 72). *In vivo* animal-model studies support a protective role of SHBG in hepatic metabolism. Higher SHBG expression downregulates hepatic ATP production and inhibit lipogenic enzymes, such as acetyl-CoA-carboxylase and fatty acid synthase, thereby reducing hepatic lipid accumulation (63, 73). Further supporting this, SHBG overexpression in transgenic mice protects against high-fat (with carbohydrate) diet (HFD)-induced obesity and insulin resistance, improving glucose tolerance, lowering insulin levels, and attenuating increases in leptin and resistin levels (63).

Importantly, the significant differences in SHBG concentrations observed between NK and SuK phases suggest that SHBG may serve as a sensitive marker of metabolic-endocrine interactions within the female hormonal axis. These findings raise important questions about whether fluctuations in SHBG, for instance as those observed from P1 (NK) to P2 (SuK) and subsequently to P3 (return to NK), reflect a compensatory response (homeostatic adaptation) or an active regulatory mechanism in hormone availability in response to altered energy metabolism, particularly under ketogenic conditions or states of metabolic dysfunction. In line with emerging literature, our data support the role of SHBG as both a proxy marker of insulin sensitivity and exposure, hepatic function, as well as a potential diagnostic biomarker and therapeutic target in the management of PCOS, MASLD, and breast cancer (13, 14). Further research is warranted to elucidate the mechanistic role of SHBG in these contexts and to evaluate its clinical relevance across broader populations.

Dietary carbohydrate restriction, particularly when inducing sustained nutritional ketosis, exerts a profound influence on SHBG concentrations and broader metabolic-endocrine signalling. Across both PCOS and non-PCOS female cohorts, ketogenic interventions that achieve euketonaemia have been consistently associated with elevations in SHBG, accompanied by improvements in glycaemic regulation, insulin sensitivity, and androgen homeostasis (18, 29–32, 68). These outcomes are driven primarily by reductions in basal circulating insulin concentrations. As insulin is the principal suppressor of hepatic ketogenesis, any degree of hyperinsulinaemia, whether overt or subclinical, can suppresses endogenous ketone generation and simultaneously inhibits SHBG synthesis within the liver (16, 34–36).

SHBG is a tightly regulated hepatic glycoprotein that binds and transports sex hormones while also serves as a sensitive biomarker of metabolic homeodynamics. Its synthesis is acutely responsive to insulin levels, thyroid hormones, and hepatic fatty acid oxidation status (19, 66, 70, 73). The restoration of higher SHBG levels through nutritional ketosis reflects a shift toward improved metabolic homeodynamics, characterised by sufficiently reduced insulin levels to allow normalisation of hepatic processing and endocrine transport dynamics. Conversely, low SHBG levels are indicative of metabolic dysfunction and actively reflect a state of insulin-excess, often preceding disturbances in reproductive hormones, glucose metabolism, and vascular tone. Thus, SHBG functions as a peripheral surrogate of chronic insulin over-exposure, with reductions in SHBG representing one of the earliest physiological responses to sustained hyperinsulinaemia and hypoketonaemia.

In this context, SHBG offers a unique window into subclinical disease states. The detection of persistently low levels of SHBG in euglycaemic individuals may signal occult hyperinsulinaemia, particularly when ketone levels remain low despite carbohydrate restriction. Such a pattern reveals hepatic insulin resistance before the onset of dysglycaemia, positioning SHBG as a frontline biomarker for the early detection of stage-1 type-2 diabetes and hypoketonaemia-ICE (38). This concept aligns with the broader framework in which chronic insulin elevation precedes and drives pathophysiological cascades implicated in cardiovascular disease, oestrogen-dependent cancers, PCOS, and neurodegeneration (35, 74–77).

When oestrogen is coupled with SHBG and both activate their respective receptors on target cells, the resulting intracellular signalling cascade differs from that initiated by oestrogen alone. Many tissues express SHBG membrane receptors, and the binding of SHBG to its receptor induces cyclic adenosine monophosphate (cAMP) activation, functioning as a signal transduction modulator. Furthermore, SHBG molecules can enter cells and exert direct intracellular effects (13, 14). SHBG downregulates the proto-oncogenes c-Myc, B-cell lymphoma-2 (Bcl-2), and growth factor receptors associated with cancer cell growth, proliferation and survival (42, 78–80). In contrast, a low SHBG setting results in cancer cells exhibiting increased levels of c-Myc expression, driving transcriptional programmes that promote cellular proliferation, aerobic fermentation, and dedifferentiation. c-Myc upregulates the glucose transporter GLUT1, enabling insulin-independent cellular glucose uptake (13, 14, 16, 74, 81–84). This enhanced capacity for glucose uptake supplies cancer cell dependence on aerobic fermentation to facilitate their bioenergetic needs via cytosolic substrate-level phosphorylation, independent of insulin mediated glucose uptake signalling (42, 80, 81, 85).

Low SHBG levels commonly observed in hyperinsulinaemic and hypoketonaemic states, result in increased c-Myc expression and activity. In cancer cells, higher levels of c-Myc expression drives increased transcription and activity of key glycolytic enzymes, including lactate dehydrogenase (LDH) and hexokinase 2 (HK2) (14, 42, 78, 80, 81). LDH plays a central role in sustaining aerobic glycolysis in proliferative tumour phenotypes by catalysing the reduction of pyruvate to lactate (80, 86–90). This reaction regenerates cytosolic nicotinamide adenine dinucleotide (NAD^+^), a redox cofactor essential for the maintenance of glycolytic flux (38, 40, 43, 56). The regeneration of NAD^+^ permits uninterrupted substrate-level phosphorylation, enabling ATP synthesis independently of mitochondrial oxidative phosphorylation (91–93). This metabolic re-routing underpins the Warburg effect, whereby cancer cells ferment glucose despite sufficient oxygen availability (42, 80). By diverting pyruvate away from mitochondrial entry, LDH functionally suppresses oxidative metabolism, concomitantly enforcing aerobic fermentation of glucose and mitochondrial substrate level phosphorylation of glutamine. This not only sustains ATP production under conditions of mitochondrial dysfunction or high biosynthetic demand but also supports anabolic precursor generation and redox homeostasis necessary for rapid cellular proliferation. The accumulation of extracellular lactate further modifies the tumour microenvironment, promoting immune evasion, angiogenesis, and stromal reprogramming.

HK2 is one of four glucokinase isoforms and catalyses the first committed step of glycolysis by phosphorylating glucose to glucose-6-phosphate, thereby trapping it within the cell. HK2 is predominantly expressed in insulin-responsive tissues, such as skeletal muscle, myocardium, and adipose tissue, where it supports postprandial glucose clearance and energy storage. However, HK2 expression is markedly upregulated in a wide range of malignant tumours, reflecting its dual regulation by insulin signalling and oncogenic drivers (94–96). Activation of the SHBG receptor decreases c-Myc levels and activity, leading to a decrease in HK2 expression. Conversely, low SHBG levels result in increased c-Myc expression, leading to increased HK2 and LDH levels (14, 81). HK2 binds directly to the outer mitochondrial membrane, where it utilises ATP generated within the mitochondrial matrix to catalyse the phosphorylation of glucose to glucose-6-phosphate. This spatial proximity, typically mediated via the voltage-dependent anion channel (VDAC), enables HK2 to bypass cytosolic ATP competition and gain privileged access to mitochondrial ATP pools. The mitochondrial anchoring of HK2 permits a high-flux glycolytic state by coupling residual oxidative phosphorylation-derived ATP to the first committed step of glucose metabolism. This arrangement not only accelerates glycolytic throughput but also insulates cancer cells from ATP depletion under fluctuating nutrient conditions. By drawing on mitochondrial energy to support cytosolic glycolysis, HK2 acts as a gatekeeper of bioenergetic partitioning, reinforcing the Warburg phenotype and promoting proliferation under oncogenic and insulin-activated signalling contexts (94, 95, 97). Hyperinsulinaemia enforces glucose oxidation over beta-oxidation and ketolysis, decreasing the cellular pool of NAD^+^. Concurrently, the mitochondrial derived ATP consumed by HK2 imposes additional mitochondrial demand of NAD^+^, as a result, mitochondrial oxidative phosphorylation is compensatorily reduced to drive up aerobic glycolysis in order to refurnish the cytosol with NAD^+^ (38, 43, 93).

Hyperinsulinaemia decreases SHBG levels, which increases c-Myc expression in cancer cells. Increased c-Myc activity, in turn, upregulates transcription and activity of LDH and HK2 (14, 81). Mitochondrial binding of HK2 inhibits Bax-induced cytochrome c release and subsequently inhibits mitochondrial death-receptor-mediated apoptosis (98). This strategic positioning enables cancer cells to integrate anabolic demands with survival pathways, reinforcing the glycolytic phenotype characteristic of aggressive, proliferative states (96).

Lower levels of SHBG receptor signalling, whilst cells receive oestrogen receptor activation, also increases Bcl-2 levels. Bcl-2 preserves cellular viability by suppressing mitochondrial apoptosis pathways at the level of the outer mitochondrial membrane, preventing the membrane permeabilisation (MOMP) required for cytochrome c release into the cytosol, thereby maintaining the integrity of the mitochondrial intermembrane space and inhibiting caspase activation (99). Bcl-2 achieves this through direct interaction with the pro-apoptotic effectors Bax and Bak, sequestering them in inactive conformations and preventing pore formation within the membrane. Through this mechanism, Bcl-2 sustains mitochondrial membrane potential (Δψm), redox balance, and ATP production, enabling the continued survival of metabolically stressed or oncogenically transformed cells.

Aggressively proliferating cancer cells overexpress Bcl-2, leading to mitochondrial resistance to apoptosis which underpins tumour persistence and therapeutic resistance. Chronic hyperinsulinaemia decreases SHBG, leading to increased Bcl-2 and c-Myc levels. c-Myc expression in cancer cells increases LDH, GLUT1, HK2, and alters transcriptional programming in transformed cells to be more similar to embryonic cells, which is a dedifferentiated, hyperplastic and hyper-prolific state (82). This convergence of metabolic and developmental reprogramming reflects mitochondrial pathophysiological transformation and the dominance of insulin–glucose–growth factor signalling loops.

Given insulin’s suppressive control over hepatic SHBG synthesis (15, 100), its measurement offers a mechanistically grounded and clinically accessible metric of metabolic endocrine status. Unlike glucose or HbA1c, which reflect late-stage dysregulation, SHBG, along with euketonaemia monitoring, provides an indirect yet precise index of the insulin axis activity and its broader metabolic-endocrine consequences. This is particularly relevant for women, in whom hypo-SHBG states are predictive of hyperandrogenism, ovulatory dysfunction, and infertility, as well as increased long-term risk for breast and endometrial cancer (1, 13, 14, 61). Importantly, SHBG levels increase rapidly in response to dietary carbohydrate restriction, reflecting improved hepatic insulin sensitivity and restoration of fatty acid oxidation (16, 34, 35, 83, 84).

Early restoration of SHBG levels via ketogenic metabolic therapy (KMT) at therapeutic levels, therefore, serves dual functions: as a marker of mechanistic reversal and as a mediator of reduced downstream hormonal and metabolic dysregulation. Its modulation is causally linked to the insulin–ketone–liver axis and should be integrated into personalised metabolic-endocrine assessments. A shift upwards in SHBG trajectory in the context of euketonaemia and reduced fasting insulin constitutes evidence of improving metabolic-endocrine health, offering an inexpensive, widely accessible surrogate for direct insulin quantification, which remains underutilised and poorly standardised in routine clinical practice (16).

SHBG plays a central regulatory role in endocrine–oncogenic signalling, with robust mechanistic evidence showing its direct modulation of bioactive sex hormone availability and intracellular receptor activation (61). Its suppression by insulin via downregulation of HNF4α links hepatic metabolic status to systemic hormonal control, establishing SHBG as a key mediator at the intersection of metabolic dysfunction, hyperinsulinaemia, and cancer risk (61). Reduced SHBG levels increase free testosterone and oestradiol, promoting tumour proliferation in hormone-responsive cancers, while elevated SHBG inhibits growth and metastasis even in receptor-negative phenotypes (13, 14, 101). These integrated findings fulfil the criteria for a central regulatory molecule and support the conclusion that SHBG functions as a protective metabolic–endocrine marker with system-wide relevance.

Thus, SHBG emerges as a pivotal indicator in the modern metabolic framework. In women, its diagnostic and prognostic relevance spans reproductive, cardiovascular, oncological, and neurological domains (19, 23, 59–61, 63, 71). In both metabolic research and clinical intervention alike, its early normalisation through maintaining euketonaemia reflects therapeutic efficacy and the re-establishment of hepatic metabolic control, preceding irreversible disease progression.

A significant elevation in circulating leptin levels was observed following the 21-day suppression of ketosis during Phase 2 (P2), characterised by sustained hypoketonaemia-ICE. Upon re-establishment of nutritional ketosis in Phase 3 (P3), leptin concentrations returned markedly toward baseline in parallel with restored euketonaemia. Linear mixed-effects modelling confirmed a strong inverse association between leptin and SHBG across phases, highlighting the shared sensitivity of both markers to shifts in insulin signalling. These findings are consistent with the role of leptin as a dynamic metabolic-endocrine biomarker of insulin tone and adipose–liver axis regulation.

Leptin, the adipocyte-derived cytokine encoded by the Ob gene, functions far beyond its canonical role in energy homeostasis, exerting pro-tumorigenic effects across multiple malignancies through mitogenic, anti-apoptotic, metabolic, and angiogenic mechanisms. Leptin promotes endothelial tube formation and increases vascular permeability by synergising with VEGF and fibroblast growth factor 2 (FGF-2), thereby facilitating tumour vascularisation and the establishment of a permissive vascular–stromal interface for cellular invasion (102–104). Within the glioblastoma microenvironment, leptin secreted by tumour cells directly stimulates endothelial growth, mirroring the effects of VEGF and sustaining tumour expansion under hypoxic and nutrient-depleted conditions (104, 105).

Leptin receptor (ObR) signalling also plays a central role in the metabolic reprogramming of cancer cells. In breast, colon, pancreatic, and endometrial cancers, leptin promotes proliferation by activating PI3K–Akt, JAK2–STAT3, MEK–ERK, and JNK signalling cascades (106–110). These cascades converge on cell cycle regulators such as cyclin D1 and Mcl-1, inhibit mitochondrial apoptotic checkpoints that are regulated by Bcl-2 and pro-apoptotic effectors (e.g., Bax, Bak), and support the preferential reliance on aerobic glycolysis over oxidative phosphorylation, driven by mitochondrial adaptation, dysfunction, and redox dysregulation. Chronic excess leptin, insulin, and IGF-1 upregulate anti-apoptotic proteins Bcl-2 and Mcl-1, whilst also inhibiting Bax/Bak oligomerisation. This stabilises the Δψm, preventing the mitochondrial outer MOMP, which inhibits the mitochondrial checkpoint that governs apoptosis initiation (109, 111). In hypothalamic astrocytes, leptin regulates nutrient transporter expression, including GLUT1 and glutamate transporters, providing further evidence of its influence on nutrient uptake and metabolic dysregulation (112).

In hormone-sensitive tissues, leptin interacts with insulin and IGF-1 signalling, forming an endocrine-oncogenic triad that supports tumour progression. Hyperglycaemia and insulin excess potentiate leptin-mediated signalling, particularly via IGF1R–Akt–mTOR activation, further accelerating cell cycle progression and biomass accumulation in breast and mammary epithelial cells (106, 113). In prostate and endometrial cancers, leptin enhances differentiation, proliferation, and invasiveness through transcriptional upregulation of proto-oncogenes and metabolic enzymes (111, 114). Long-term exposure to leptin increases tumour cell viability and shifts the mitochondrial phenotype toward apoptosis evasion and aerobic glycolysis, enabling anabolic growth in nutrient-variable microenvironments (115–119).

Leptin’s role in metastasis is also increasingly recognised. In pancreatic and ovarian cancers, leptin–ObR signalling promotes extracellular matrix remodelling, migration, and invasion through upregulation of matrix metalloproteinase-13, an enzyme that breaks down the extracellular matrix, particularly collagen, and downstream effectors (110, 120). In ovarian cancer cells, leptin maintains stem-like characteristics and drives a more aggressive transcriptional phenotype, explaining the poor prognosis observed in obese patients with leptin resistance or chronic hyperleptinaemia (121, 122). Clinical data further associate high leptin levels with increased prostate cancer risk and unfavourable outcomes in ovarian cancer (122–124).

Leptin and its receptor ObR are overexpressed in malignant brain tumours and display a strong positive correlation with histopathological grade, with the highest levels consistently observed in glioblastoma (GBM), a WHO grade IV astrocytic neoplasm (105). This overexpression actively contributes to tumour pathophysiology. Leptin engages proliferative, anti-apoptotic, and migratory signalling via PI3K–Akt and JAK2–STAT3 cascades, thereby supporting cellular survival within the tumour microenvironment. These signalling axes converge with broader patterns of metabolic reprogramming, redox mal-adaptation, and downregulation of mitochondrial oxidative metabolism, enabling sustained proliferation even under hypoxic or nutrient-depleted conditions (104, 105, 107, 111, 113, 115, 116, 125–129).

Functioning additionally as a pro-angiogenic cytokine, leptin promotes the expression of VEGF and stimulates endothelial tube formation, driving neovascularisation in support of tumour expansion (34, 35, 103, 104, 130). This angiogenic phenotype is especially prominent in GBM, where vascular proliferation ensures perfusion and also establishes a permissive vascular–stromal interface for cellular migration and infiltration. Notably, GBM cells possess the capacity for autocrine leptin production, allowing locally secreted leptin to activate ObR on neighbouring or self-same tumour cells. This autocrine loop amplifies survival signalling, suppresses mitochondrial apoptotic checkpoints, and facilitates the autonomous maintenance of ATP production, redox cycling, and anabolic flux, independent of systemic endocrine input (104, 105, 131).

Within the broader metabolic–endocrine context, hyperinsulinaemia and suppression of SHBG frequently co-occur with leptin resistance, wherein elevated leptin reflects compensatory hypersecretion rather than receptor activity (132–135). In glioblastoma, the leptin–ObR axis actively contributes to tumour metabolism, regulating glutamate and glucose transporters, reinforcing reliance on aerobic glycolysis and glutaminolysis over oxidative phosphorylation, driven by mitochondrial adaptation and dysfunction (105, 112, 131). These endocrine–oncogenic dynamics support the therapeutic rationale for KMT for cancer. By raising SHBG, improving leptin sensitivity, and decreasing excess insulin-mediated signalling, nutritional and therapeutic ketosis may attenuate this endocrine, paracrine and autocrine growth axis and re-sensitise tumour cells to apoptotic initiation (16, 34, 35). Targeting the metabolic dependencies that sustain tumour bioenergetics offers a viable adjunct to existing therapeutic modalities. Based on KMT’s multiple mechanisms of action, this provides a rational explanation of how KMT would improve patient responses to standard of care (SOC) oncology treatments, including chemotherapy, immunotherapy, checkpoint inhibitors, receptor inhibitors, immune cell-based vaccines and radiotherapy.

Leptin, thus, acts as a pleiotropic mitokine, coordinating energy-sensing, redox adaptation, apoptosis resistance, and vascular remodelling within the tumour microenvironment. Its downstream effects reinforce the oncogenic landscape established by chronic hyperinsulinaemia, hypoketonaemia and low SHBG. This amplifies proliferative and invasive capacity while inhibiting apoptosis. Therapeutic strategies targeting leptin–ObR signalling may restore mitochondrial sensitivity to redox stress and re-establish endocrine regulation, particularly when combined with ketogenic endocrine metabolic oncology (KEMO) therapies that decrease insulin signalling, increase SHBG and euketonaemia (16, 34, 35, 41, 42, 136).

Beyond SHBG and leptin, we also investigated the effects of ketosis suppression on key reproductive hormones, including oestrogen, progesterone, testosterone, LH, and FSH. While no significant changes were detected in circulating levels of oestrogen, progesterone, testosterone, LH, or FSH across the study phases and in linear effects models, FSH concentrations varied significantly by menstrual cycle phase, with elevated levels during ovulation compared to the follicular phase. This aligns with the physiological role of FSH in follicular development and emphasises the importance of accounting for cycle phase in hormonal-endocrine research. These findings are particularly relevant, given the increasing interest in how dietary strategies, such as low-carbohydrate or ketogenic diets, influence female reproductive physiology. While carbohydrate restriction has shown therapeutic benefits in PCOS, characterised by hyperandrogenism and ovulatory dysfunction, its effects in healthy, reproductive-age women remain underexplored. The present data contribute to this emerging field by demonstrating the apparent hormonal resilience of the reproductive axis in response to short-term changes in metabolic state.

### 4.1 Strengths and limitations

This study offers a novel investigation into female hormone-related key biomarkers within the context of metabolic health and ketogenic metabolic therapy in a cohort of healthy premenopausal women adapted to long-term nutritional ketosis. A major strength is the within-subject crossover design, which allowed for metabolically distinct phase comparisons and improved internal validity. The cohort’s prolonged keto-adaptation offers unique insights into chronic ketosis’ influence on females’ endocrine regulation. However, further studies in larger, more diverse populations, including males, older adults, both keto-adapted and non-keto-adapted, and individuals with metabolic disorders or chronic health conditions, such as PCOS, across a wider age range and health spectrum, are warranted to validate and extend our findings.

Although the menstrual cycle stage did not significantly affect most measured biomarkers in this study, a more comprehensive analysis across cycle stages is planned in future publications to clarify cycle-dependent variability, particularly in relation to systemic biomarkers beyond female sex hormones. Such analyses will enhance our understanding of physiological fluctuations and improve biomarker interpretation in premenopausal women. Additional methodological considerations, including power calculations and sample size estimations, have been detailed in our previous work (16, 34, 35, 41).

## 5 Conclusion

This study represents the first controlled investigation into the effects of long-term sustained nutritional ketosis, and its deliberate suppression, on SHBG and related metabolic–reproductive signalling in healthy premenopausal women. These findings establish SHBG as a dynamic biomarker and regulator within the insulin–liver axis, integrating metabolic and reproductive signalling and demonstrating responsiveness to changes in dietary carbohydrate load. Restoration of euketonaemia, achieved through carbohydrate restriction, whilst consuming adequate natural healthy fats, consistently increased SHBG levels, indicating improved hepatic insulin sensitivity and reversal of insulin-driven endocrine suppression. Importantly, this effect occurred without destabilising reproductive hormone profiles, supporting the safety of carbohydrate restriction in premenopausal women. The elevation of SHBG through therapeutic ketosis holds particular relevance for oncology. In multiple tumour contexts, low SHBG states are associated with increased c-Myc and Bcl-2 expression and activity, upregulation of glycolytic effectors GLUT1, LDH and HK2, promoting mitochondrial resistance to apoptosis.

Chronic hyperinsulinaemia suppresses hepatic SHBG synthesis, thereby disinhibiting these oncogenic pathways, driving increased aerobic glycolysis and substrate level phosphorylation dependence, proliferative signalling, and apoptosis resistance. Elevated leptin, under insulin resistance and hyperinsulinaemia, further reinforces this oncogenic landscape by activating mitogenic, anti-apoptotic, and pro-angiogenic pathways (JAK2-STAT3 and PI3K-Akt) and maintaining cancer stem-like phenotypes in many tumour types. Elevation of SHBG through euketonaemia, acts as a counter-regulatory signal that downregulates substrate level phosphorylation dependence and restores apoptotic capacity. These findings support the therapeutic rationale for KMT in cancer, where correction of hyperinsulinaemia and induction of euketonaemia restricts tumour fuel supply, thus reprogramming endocrine-oncogenic signalling through SHBG modulation. Further translational studies are warranted to determine whether SHBG normalisation may serve as both a biomarker and a mediator of therapeutic response in KMT-treated cancer patients. This paper identifies SHBG as a central regulator of endocrine–oncogenic signalling, with elevated SHBG functioning as a protective metabolic-endocrine marker. Insulin thus drives the cancer cell phenotype via transcriptional reprogramming and downregulation of mitochondrial apoptosis capability. Leptin, likewise, regulated by insulin and nutritional status, reinforces this oncogenic landscape. Leptin levels rise under insulin resistance and hyperinsulinaemia, amplifying proliferative signalling and further decoupling tumour growth from endocrine restraint.

Finally, this work also advances a refined diagnostic framework for hyperinsulinaemia. We propose that persistent suppression of BHB below 0.5 mmol/L before the evening meal, despite normoglycaemia and reference-range insulin, indicates hypoketonaemia-Insulin-Compensated Euglycaemia (ICE), a subclinical state of pathological insulin excess. This functional phenotype, linked to the Personal Hyperinsulinaemia Threshold (PIT), is routinely missed by conventional glucose-centric assessments. By integrating SHBG with fasting insulin, leptin, and ketone-based metabolic phenotyping, both research and clinical frameworks can achieve earlier detection of SCHI. SHBG and leptin, when interpreted in context, provide mechanistically grounded biomarkers that support timely metabolic intervention before the onset of pathology such as cancer progression.

## Data Availability

The original contributions presented in the study are included in the article/supplementary material. Further inquiries can be directed to the corresponding author.

## Glossary

Bcl-2: B-cell lymphoma-2
BHB: Beta-hydroxybutyrate
BIA: Bioelectrical impedance
BMI: Body mass index
cAMP: Cyclic adenosine monophosphate
CoA: Coenzyme A
CVD: Cardiovascular disease
EDTA: Ethylenediaminetetraacetic acid
ELISA: Enzyme-linked immunosorbent assay
Free T3: Free triiodothyronine
FSH: Follicle-stimulating hormone
GBM: Glioblastoma
GGT: Gamma-glutamyl transferase
GKI: Glucose ketone index
GLP-1: Glucagon-like peptide 1
HbA1c: Haemoglobin A1c
HFD: High fat diet (with carbohydrate)
HK2: Hexokinase 2
HOMA-IR: Homeostasis model assessment for insulin resistance
ICE: Insulin-Compensated Euglycaemia
Idh2: Isocitrate dehydrogenase 2
IGF-1: Insulin-like growth factor 1
IR: Insulin resistance
KEMO: Ketogenic endocrine metabolic oncology
KMT: Ketogenic metabolic therapy
LDH: Lactate dehydrogenase
LH: Luteinising hormone
MASLD: Metabolic-dysfunction-associated steatosis liver disease
MetS: Metabolic syndrome
MOMP: Membrane permeabilisation
NAD^+^: Nicotinamide adenine dinucleotide
NK: Nutritional ketosis
ObR: Leptin receptor
OGTT: Oral glucose tolerance test
PCOS: Polycystic ovarian syndrome
PIT: Personalised hyperinsulinaemia threshold
P1: Phase 1
P2: Phase 2
P3: Phase 3
RM: Repeated measures
RQ: Respiratory quotient
SCHI: subclinical hyperinsulinaemia
SHBG: Sex hormone-binding globulin
SOC: Standard of care
SuK: Suppression of ketosis
T2DM: Type 2 diabetes mellitus
T4: Thyroxine
TSH: Thyroid stimulating hormone
VDAC: Voltage-dependent anion channel
VEGF: Vascular endothelial growth factor
WHO: World Health Organisation
*β*: Effect estimate
Δψm: Mitochondrial membrane potential
